# Patient-reported outcomes after oesophagectomy in the multicentre LASER study

**DOI:** 10.1093/bjs/znab124

**Published:** 2021-05-11

**Authors:** S R Markar, V Sounderajah, A Johar, G Zaninotto, C Castoro, P Lagergren, J A Elliott, S S Gisbertz, C Mariette, R Alfieri, J Huddy, E Pinto, M Scarpa, F Klevebro, B Sunde, C F Murphy, C Greene, N Ravi, G Piessen, H Brenkman, J Ruurda, R van Hillegersberg, S M Lagarde, B P Wijnhoven, M Pera, J Roigg, S Castro, R Matthijsen, J Findlay, S Antonowicz, N Maynard, O McCormack, A Ariyarathenam, G Sanders, E Cheong, S Jaunoo, W Allum, J van Lanschot, M Nilsson, J V Reynolds, M I van Berge Henegouwen, G B Hanna

**Affiliations:** 1 Department Surgery and Cancer, Imperial College London, London, UK; 2 Division of Surgery, Department of Clinical Science Intervention and Technology (CLINTEC), Karolinska Institutet, Stockholm, Sweden; 3 Department of Upper Abdominal Diseases, Karolinska University Hospital, Stockholm, Sweden; 4 Unit of Surgical Oncology of the Oesophagus and Digestive Tract, Veneto Institute of Oncology, Padua, Italy; 5 Department of Surgery, Trinity Centre for Health Sciences, St James’s Hospital and Trinity College Dublin, Dublin, Ireland; 6 Department of Surgery, Cancer Centre Amsterdam, Amsterdam UMC, location AMC, University of Amsterdam, Amsterdam, the Netherlands; 7 Department of Digestive and Oncological Surgery, University of Lille, Claude Huriez University Hospital, F-59000 Lille, France; 8 Department of Surgery, University Medical Centre Utrecht, Utrecht, the Netherlands; 9 Department of Surgery, Erasmus MC, University Medical Centre, Rotterdam, the Netherlands; 10 Department of Surgery, University Hospital del Mar, Barcelona, Spain; 11 Department of Gastrointestinal Surgery, ETZ Tildburg, Tildburg, the Netherlands; 12 Oesophago-gastric Centre, Churchill Hospital, University of Oxford, Oxford, UK; 13 Department of Oesophago-Gastric Surgery, Royal Marsden Hospital, London, UK; 14 Department of Oesophago-Gastric Surgery, Plymouth Hospitals NHS Trust, Plymouth, UK; 15 Department of Upper Gastrointestinal Surgery, Norfolk and Norwich Hospitals NHS Trust, Norwich, UK; 16 Department of Upper Gastrointestinal Surgery, Gloucestershire Hospitals NHS Foundation Trust, Gloucester, UK

## Abstract

**Background:**

Data on the long-term symptom burden in patients surviving oesophageal cancer surgery are scarce. The aim of this study was to identify the most prevalent symptoms and their interactions with health-related quality of life.

**Methods:**

This was a cross-sectional cohort study of patients who underwent oesophageal cancer surgery in 20 European centres between 2010 and 2016. Patients had to be disease-free for at least 1 year. They were asked to complete a 28-symptom questionnaire at a single time point, at least 1 year after surgery. Principal component analysis was used to assess for clustering and association of symptoms. Risk factors associated with the development of severe symptoms were identified by multivariable logistic regression models.

**Results:**

Of 1081 invited patients, 876 (81.0 per cent) responded. Symptoms in the preceding 6 months associated with previous surgery were experienced by 586 patients (66.9 per cent). The most common severe symptoms included reduced energy or activity tolerance (30.7 per cent), feeling of early fullness after eating (30.0 per cent), tiredness (28.7 per cent), and heartburn/acid or bile regurgitation (19.6 per cent). Clustering analysis showed that symptoms clustered into six domains: lethargy, musculoskeletal pain, dumping, lower gastrointestinal symptoms, regurgitation/reflux, and swallowing/conduit problems; the latter two were the most closely associated. Surgical approach, neoadjuvant therapy, patient age, and sex were factors associated with severe symptoms.

**Conclusion:**

A long-term symptom burden is common after oesophageal cancer surgery.

## Teaser

The aim of this questionnaire study was to identify long-term symptoms that affect quality of life after oesophageal cancer surgery. Symptoms clustered into six domains: lethargy, musculoskeletal pain, dumping, lower gastrointestinal symptoms, regurgitation/reflux, and swallowing problems. This demonstrates a poorly appreciated long-term symptom burden.

## Introduction

Surgical resection is still the mainstay of curative treatment for oesophageal cancer. Centralization of oesophageal cancer surgery^1^, implementation of enhanced recovery protocols^2^, and adoption of minimally invasive techniques^3^ have reduced morbidity and treatment-related mortality. Multimodal treatment has led to further improved survival rates^4^^,^^5^. Incremental improvements in long-term survival have highlighted the increasing importance of quantifying long-term health-related quality of life (HRQoL) of oesophageal cancer survivors^6^ using validated disease-specific tools.

Validated questionnaires for the assessment of HRQoL during treatment of oesophageal cancer have been produced by the European Organization for Research and Treatment of Cancer (EORTC)^7^^,^^8^. These have been used to assess the impact of multimodal treatment^9^, and the effect of variation in surgical technique^10^ and complication burden, on HRQoL. These questionnaires have, however, not been validated for assessment of HRQoL in the disease-free survivorship state. Long-term persistent adverse effects of oesophageal cancer surgery on disease-free HRQoL have been described^11^, but a robust assessment of HRQoL in the context of contemporary treatment strategies is lacking. A recent population-based cohort study^12^ from England suggested that over 40 per cent of patients visit their primary-care physician after oesophagectomy with long-term symptoms as a result of the intervention. The presence of two or more symptoms was associated with an increased risk of new-onset depression or anxiety.

The multicentre LASER (LAsting Symptoms after Esophageal Resection) study was developed to evaluate the burden of long-term symptoms in survivors more than 1 year after initial treatment^13^. The aim was to identify the most prevalent symptoms affecting HRQoL, to establish whether symptoms cluster into domains, and to identify potential patient and treatment factors associated with development of long-term symptoms.

## Methods

###  

This was a multicentre study of consecutive patients who underwent oesophageal cancer surgery at 20 European centres between 1 January 2010 and 30 June 2016. Patients who had oesophagectomy with curative intent for either oesophageal or junctional (Siewert I and II) cancer and who were disease-free for at least 1 year after completion of initial treatment, either surgery or adjuvant therapy, were eligible. They were asked to complete the validated LASER questionnaire on a single occasion.

Patients who underwent salvage oesophagectomy after initial endoscopic or oncological treatment with persistent or recurrent oesophageal cancer were also included. Exclusion criteria were: endoscopic therapy or definitive chemoradiotherapy as sole therapy for oesophageal cancer, known cancer recurrence, ongoing surgical complications (such as tracheostomy), and non-oral nutrition (for example, jejunostomy catheter).

Local institutional review board and ethical approvals were obtained by each participating centre.

### Intervention

The LASER questionnaire was used to evaluate the presence and severity of 28 symptoms in the 6 months preceding administration of the survey^13^. The development and collection of the LASER data set have been described previously^13^. In summary, the LASER questionnaire was developed through a Delphi consensus process of clinician experts in addition to input from international patient workshops. This questionnaire was used to collect data from all patients in the LASER study (*Appendix S1*) via either an electronic or paper format. These results were subsequently uploaded to a specifically created website (https://www.laserstudy.org) for secure data storage and analysis.

Each symptom from the LASER questionnaire was graded according to its impact on quality of life, measured by means of validated EORTC QLQ-C30^6^ and QLQ-OG25^7^ questionnaires, and the frequency of the symptom was recorded, using a composite score from 0 to 5 (*Table S1*). A symptom grade of 3 or higher was indicative of either a frequent symptom or a symptom with a substantial impact on quality of life; these were therefore classified as severe symptoms.

**Table 1:** Demographics and clinicopathological data for patients in the LASER study

	No. of patients*
**Age (years)**†	65 (58–70)
**Sex**	
M	559 (77.1)
F	166 (22.9)
Missing	56
**Weight (kg)**†	78 (68–88)
**Access to chest**	
Ivor Lewis	450 (57.6)
Left thoracoabdominal	48 (6.2)
McKeown	175 (22.4)
Transhiatal	108 (13.8)
**Surgical approach**	
Open	392 (54.3)
Hybrid minimally invasive‡	134 (18.6)
Totally minimally invasive	196 (27.2)
Missing	59
**Anastomosis site**	
Cervical	283 (39.2)
Thoracic	439 (60.8)
Missing	59
**Anastomosis orientation**	
End to end	200 (28)
End to side	464 (64.9)
Side to side	51 (7.1)
Missing	66
**Pyloric drainage**	
Drainage procedure	236 (33)
No drainage	479 (67)
Missing	66
**Postoperative feeding jejunostomy**	
No	160 (23.3)
Yes	527 (76.7)
Missing	94
**Neoadjuvant therapy**	
No	148 (20.7)
Yes	567 (79.3)
Missing	66
**Adjuvant therapy**	
No	532 (84)
Yes	101 (16)
Missing	148
**TNM stage (7th edition)**	
0	138 (20)
I	225 (32.7)
II	174 (25.3)
III–IV	152 (22.1)
Missing	92
**30-day postoperative complication**	
No	311 (42.9)
Yes	414 (57.1)
Missing	56
**Clavien–Dindo grade**	
0	311 (39.8)
I	42 (5.4)
II	187 (23.9)
III	125 (16.0)
IV	46 (5.9)
Missing	70
**Country**	
Netherlands	223 (28.8)
UK	245 (31.5)
Sweden	74 (9.5)
France	33 (4.2)
Italy	93 (12)
Ireland	75 (9.6)
Spain	34 (4.4)
Missing	3

*With percentages in parentheses unless indicated otherwise; †values are median (i.q.r.).

‡ Laparoscopic abdomen and open chest, or open abdomen and thoracoscopic chest.

**Table 2:** Results of principal component analysis in classification of six symptom clusters

Symptom cluster	Symptoms included
Lethargy	Lack of appetite Tiredness Low mood Reduced energy or activity tolerance Abnormal sensation in fingers and toes
Musculoskeletal pain	Chest pain Pain from scars on chest Pain from scars on abdomen
Dumping	Abdominal pain Heart palpitations after eating Sweating after eating Dizziness after eating Bloating or cramping after eating
Lower gastrointestinal	Loose bowel motions/diarrhoea after eating Stools that float or are difficult to flush Diarrhoea unrelated to eating
Regurgitation/reflux	Regurgitation of food Nausea Vomiting Heartburn, acid/bile regurgitation Waking up during the night because of choking sensation Persistent cough Dental problems Voice problems
Swallowing/conduit symptoms	Difficulty getting food down Difficulty getting liquids down Early feeling of fullnessHiccoughs

### Data collection

In addition to collection of data on quality of life, clinicians at participating centres were asked to provide additional clinical, pathological, and demographic metadata (full list provided in *Appendix S2*).

### Statistical analysis

Basic frequency analysis was undertaken to identify the prevalence of severe symptoms. Principal component analysis was used to establish whether symptoms clustered into specific domains and whether these domains were associated. Multivariable logistic regression models were constructed to identify patient and clinical risk factors associated with the development of specific severe long-term symptoms (dependent variable). The independent variables included in these models were patient age (continuous), sex (female or male), time since surgery (continuous), neoadjuvant therapy (yes or no), surgical approach (open, hybrid minimally invasive oesophagectomy or totally minimally invasive oesophagectomy), access to the chest (transhiatal, Ivor Lewis, three-stage, or left thoracoabdominal), and postoperative 30-day complications (yes or no). Bonferroni correction was used to adjust for the effects of interaction between variables and multiple testing effects.

All statistical analyses were conducted by an experienced biostatistician using SAS^®^ version 9.4 (SAS institute, Cary, North Carolina, USA). *P* < 0.050 was considered statistically significant.

## Results

A total of 876 of 1081 invited patients (81.0 per cent) responded to the questionnaire.

Of these, 586 (66.9 per cent) stated they had experienced symptoms in the past 6 months associated with the oesophagectomy. They filled out the questionnaire a median of 4.3 (i.q.r. 3.1–5.8)  years after surgery. Patient and clinical characteristics are summarized in *Table 1*.

### Clustering, frequency and severity of symptoms

**Fig. 1:**
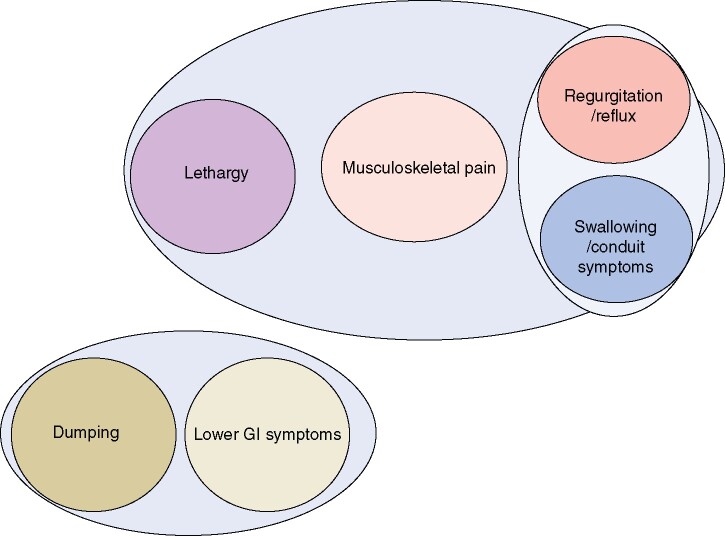
Clustering analysis showing six distinct domains of symptom clustering GI, gastrointestinal.

The 28 symptoms from the LASER questionnaire appeared to cluster into six key domains: lethargy, musculoskeletal pain, dumping, lower gastrointestinal symptoms, regurgitation/reflux, and swallowing/conduit problems (*Tables 2* and *3*) (*Fig. S1*). Regurgitation/reflux symptoms and swallowing/conduit problems were closely associated, as were dumping and lower gastrointestinal symptoms (*Fig. 1*).

**Table 3:** Constituent variables within each symptom cluster in addition to their respective proximity measures

	R^2^ with			1-R**^2^ ratio
	Own cluster	**Next** **closest**		
**Cluster 1**				
Lack of appetite	0.491	0.213		0.646
Tiredness	0.669	0.266		0.451
Low mood	0.573	0.177		0.518
Reduced energy or activity tolerance	0.601	0.243		0.526
Abnormal sensation in fingers and toes	0.291	0.105		0.792
Dental problems	0.201	0.073		0.861
**Cluster 2**				
Abdominal pain	0.450	0.202		0.689
Heart palpitations after eating	0.562	0.114		0.494
Sweating after eating	0.632	0.122		0.419
Dizziness after eating	0.540	0.169		0.554
Bloating or cramping after eating	0.481	0.243		0.686
**Cluster 3**				
Loose bowel motions/diarrhoea after eating	0.709	0.152		0.344
Stools that float or are difficult to flush	0.567	0.108		0.485
Diarrhoea (> 3 times per day) unrelated to eating	0.649	0.081		0.382
**Cluster 4**				
Chest pain	0.410	0.195		0.733
Pain from scars on chest	0.672	0.093		0.361
Pain from scars on abdomen	0.596	0.105		0.452
Voice problems	0.292	0.081		0.770
**Cluster 5**				
Regurgitaion of food	0.579	0.262		0.567
Nausea	0.500	0.274		0.688
Vomiting	0.5978	0.194		0.499
Heatburn, acid/bile regurgitation	0.515	0.154		0.5730
Waking up during the night because of choking sensation	0.418	0.155		0.689
Persistent cough	0.316	0.116		0.774
**Cluster 6**				
Difficulty getting food down	0.710	0.271		0.400
Difficulty getting liquids down	0.596	0.196		0.502
Early feeling of fullness after eating	0.520	0.250		0.640
Hiccoughs	0.244	0.093		0.834

**Table 4:** Multivariable analysis of clinical factors associated with development of the most common severe symptoms (prevalence of at least 15 per cent in the study population)

	Odds ratio			
	**Redu**ce**d energy or activity tolerance**	Early feeling of fullness after eating	Tiredness	Heartburn/acid/bile regurgitation
**Age (per year)**	1.01 (0.99, 1.03)	1.02 (1.00, 1.04)	1.03 (1.01, 1.05)	1.03 (1.01, 1.06)
**Sex**				
M	1.00 (reference)	1.00 (reference)	1.00 (reference)	1.00 (reference)
F	1.07 (0.72, 1.60)	0.73 (0.49, 1.08)	0.77 (0.52, 1.16)	0.60 (0.39, 0.94)
**Time since surgery (per year)**	0.99 (0.89, 1.10)	1.08 (0.97, 1.20)	1.03 (0.93, 1.15)	0.99 (0.88, 1.12)
**Neoadjuvant therapy**				
No	1.00 (reference)	1.00 (reference)	1.00 (reference)	1.00 (reference)
Yes	1.04 (0.69, 1.56)	1.19 (0.79, 1.78)	1.62 (1.08, 2.43)	1.18 (0.73, 1.91)
**Surgical access**				
Open	1.00 (reference)	1.00 (reference)	1.00 (reference)	1.00 (reference)
Hybrid minimally invasive	1.13 (0.70, 1.84)	1.66 (0.99, 2.76)	1.14 (0.70, 1.87)	1.03 (0.57, 1.86)
Totally minimally invasive	0.63 (0.42, 0.93)	0.87 (0.59, 1.29)	0.87 (0.58, 1.31)	0.66 (0.41, 1.06)
**Surgical technique**				
Transhiatal	1.00 (reference)	1.00 (reference)	1.00 (reference)	1.00 (reference)
Ivor Lewis	1.49 (0.92, 2.42)	1.53 (0.95, 2.49)	1.02 (0.61, 1.69)	1.05 (0.58, 1.91)
Left thoracoabdominal	1.22 (0.56, 2.68)	1.17 (0.54, 2.50)	1.17 (0.51, 2.68)	0.55 (0.23, 1.30)
Three stage	1.17 (0.69, 1.99)	1.32 (0.77, 2.25)	1.00 (0.57, 1.76)	1.05 (0.54, 2.01)
**Complications**				
No	1.00 (reference)	1.00 (reference)	1.00 (reference)	1.00 (reference)
Yes	0.81 (0.58, 1.14)	0.79 (0.56, 1.11)	0.94 (0.66, 1.33)	1.21 (0.81, 1.81)

Values in parentheses are 95 per cent confidence intervals.

**Fig. 2:**
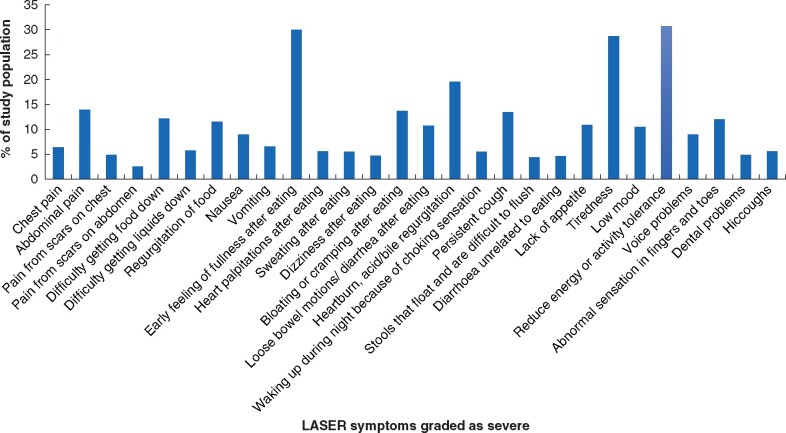
Prevalence of LASER symptoms graded as severe (score at least 3)

**Fig. 3:**
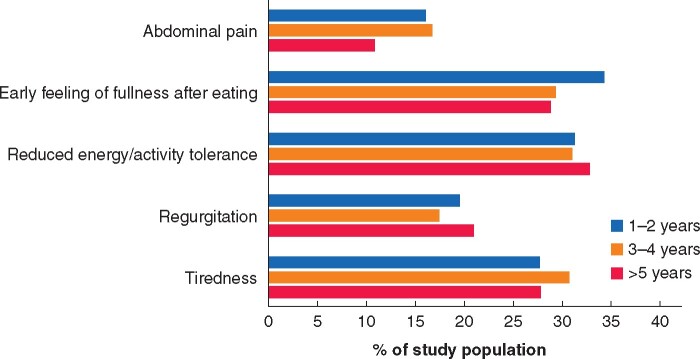
Change in profile over time of the five most commonly cited symptoms

The symptoms most commonly classified as severe were reduced energy or activity tolerance (30.7 per cent), early satiety (30.0 per cent), tiredness (28.7 per cent), heartburn/acid or bile regurgitation (19.6 per cent), abdominal pain (14.0 per cent), bloating or cramping after eating (13.7 per cent), persistent cough (13.4 per cent), and difficulty getting food down (12.2 per cent) (*Fig. 2*). An early feeling of fullness after eating was noted to be the most common symptom in the first 2 years after surgery (34.4 per cent). Reduced energy or activity tolerance was the most common symptom 3–4 years after surgery (31.1 per cent) as well as beyond 5 years (32.9 per cent) (*Appendix S3*). *Fig. 3* also highlights the dynamic nature of symptom progression over time, focusing on the five most prevalent symptoms.

### Factors associated with symptoms

Results of multivariable analysis addressing the impact of demographics and clinicopathological characteristics on severe symptoms are available in *Appendix S4*. Results for the most prevalent four severe symptoms with a prevalence of least 15 per cent are shown in *Table 4*.

A totally minimally invasive approach to oesophagectomy was associated with a lower prevalence of reduced energy or activity tolerance, increased chest pain, loose bowel motions or diarrhoea after eating, dental problems, and reduced vomiting.

Each surgical technique (Ivor Lewis, McKeown, transhiatal, left thoracoabdominal) was associated with unique symptom profiles.

The presence of 30-day postoperative complications was associated with increased prevalence of severe chest pain.

Neoadjuvant therapy was associated with an increased prevalence of tiredness.

Increasing patient age was associated with increased early feeling of fullness after eating, tiredness, and heartburn/acid or bile regurgitation, increased prevalence of abdominal pain, pain from scars on the chest, difficulty in getting food down, regurgitation of food, nausea, vomiting, heart palpitations after eating, sweating after eating, bloating or cramping after eating, loose bowel motions or diarrhoea after eating, waking up during the night because of choking sensation, and diarrhoea unrelated to eating.

Female sex was associated with a reduced prevalence of heartburn/acid or bile regurgitation, reduced prevalence of chest pain, nausea, vomiting, heart palpitations after eating, sweating after eating, bloating or cramping after eating, loose bowel motions or diarrhoea after eating, waking up during the night because of choking sensation, and diarrhoea unrelated to eating.

## Discussion

Two-thirds of patients experienced symptoms related to oesophagectomy more than 1 year after surgery. The most common severe symptoms were reduced energy or activity tolerance, early feeling of fullness after eating, tiredness, and heartburn/acid or bile regurgitation. This evidence about symptom frequency complements previous findings regarding symptoms that have the greatest impact on quality of life^13^. Symptoms tended to cluster into six domains. Choice of surgical approach, sex, and patient age were all factors in determining the symptom burden following treatment.

The extent of the hidden survivorship burden that patients in this cohort faced, even in their supposedly disease-free state, deserves comment. Reduced energy/activity tolerance and tiredness are well established associative factors in the development of major depressive episodes, which in turn have been linked with poor survival outcomes^14^. There is a growing body of survivorship-specific evidence among a range of cancers to suggest that chronic unattended symptoms have a sizeable impact on the HRQoL of cancer survivors^15^. This challenges healthcare professionals to address symptoms that they may perceive as benign during clinical consultations in order to prevent downstream effects that can manifest as poor patient outcomes.

This study may aid healthcare professionals in conceptualizing how these symptoms fit into symptom phenotypes. Knowledge of how symptoms cluster into domains, particularly when combined with personalized patient and clinical risk factor susceptibilities, will help to accurately stratify and predict survivorship care needs at an individual level^16^. Knowledge of modifiable (such as surgical approach) and non-modifiable (for example, age and sex) risk factors in the development of long-term symptoms may help in creation of personalized care plans.

Many specialist centres currently lack dedicated follow-up survivorship programmes. As such, issues including health^17^ and psychological well-being promotion, particularly in at-risk populations, are not managed systematically. Further effort should be made to counsel patients appropriately in the pretreatment phase. Future steps should include mechanistic investigation of symptom clusters in order to develop tailored interventions that may improve the long-term burden of survivorship. Specific analyses should include investigation of changes in gut hormone profiles^18^ and the microbiome. Chest wall and pulmonary dynamic investigations alongside physical activity assessments will be invaluable in understanding how the effects of oesophagectomy may be linked to reduced energy symptom profiles.

Strengths of the present study include the response rate, the multicentre international design, use of a refined and validated questionnaire, and a set-up allowing patients to complete the questionnaire outside the clinical setting.

This study had several limitations. The cross-sectional design, centred around a single episode of data collection after surgery, precluded comparison with preoperative baseline HRQoL and may have caused bias as patients did not fill out the form at a similar point in time after treatment. It is impossible to study longitudinal changes in symptoms adequately within the study design. Patients with recurrences were not included. BMI was recorded poorly in the data set. Input into the study from predominantly high-volume Western European centres may mean that the findings have limited generalizability to patients undergoing interventions in lower-volume centres or in other parts of the world.

## Funding

S.R.M. received a European Society for Medical Oncology Clinical Research Fellowship to support this study. S.R.M. is supported by a National Institute for Health Research (NIHR) Academic Clinical Lectureship and acknowledges support from the NIHR Imperial Biomedical Research Centre. This study was also supported by the NIHR London In Vitro Diagnostics (IVD) Co-operative and the Morgagni Charity. The views expressed are those of the authors and not necessarily those of the National Health Service, the NIHR or the Department of Health.

*Disclosure*. The authors declare no conflict of interest.

## Supplementary material

Supplementary material is available at *BJS* online.

## Supplementary Material

znab124_Supplementary_Data
