# Supplementary material for: Patient-reported outcomes after oesophagectomy in the multicentre LASER study
Source: Br J Surg. 2021 May 11;108(9):1090–6. doi: 10.1093/bjs/znab124 (PMC10364861; doi:10.1093/bjs/znab124)
Supplement: znab124_Supplementary_Data [file znab124_supplementary_data.zip › Appendix S1_LASER questionnaire.docx]

**Appendix S1 – LASER Questionnaire**

| **Question** | **Answer** |
| --- | --- |
| Q1. What is your current age? |  |
| Q2. What is your sex?  (Male/Female) |  |
| Q3. In the last 6 months have you had any symptoms that you associated with your oesophagectomy?  (Yes / No) |  |

|  | **Q4. Do you have any of the following symptoms and how often? Please mark** | | | | |  | **Q5. What is the impact of these symptoms to your quality of life?** | | |
| --- | --- | --- | --- | --- | --- | --- | --- | --- | --- |
| **Symptom** | **Never** | **Rarely** | **Weekly** | **Daily** | **Multiple times per day** |  | **None** | **Some** | **Substantial** |
| a) Chest pain |  |  |  |  |  |  |  |  |  |
| b) Abdominal pain |  |  |  |  |  |  |  |  |  |
| c) Pain from scars on your chest |  |  |  |  |  |  |  |  |  |
| d) Pain from scars on your abdomen |  |  |  |  |  |  |  |  |  |
| e) Difficulty getting food down |  |  |  |  |  |  |  |  |  |
| f) Difficultly getting liquids down |  |  |  |  |  |  |  |  |  |
| g) Regurgitation of food |  |  |  |  |  |  |  |  |  |
| h) Nausea |  |  |  |  |  |  |  |  |  |
| i) Vomiting |  |  |  |  |  |  |  |  |  |
| j) Early feeling of fullness after eating |  |  |  |  |  |  |  |  |  |
| k) Heart palpitation after eating |  |  |  |  |  |  |  |  |  |
| l) Sweating after eating |  |  |  |  |  |  |  |  |  |
| **Symptom** | **Never** | **Rarely** | **Weekly** | **Daily** | **Multiple times per day** |  | **None** | **Some** | **Substantial** |
| m) Dizziness after eating |  |  |  |  |  |  |  |  |  |
| n) Bloating or cramping after eating |  |  |  |  |  |  |  |  |  |
| o) Loose bowel motions / diarrhea after eating |  |  |  |  |  |  |  |  |  |
| p) Heartburn/acid/bile (sour/bitter tasting) regurgitation |  |  |  |  |  |  |  |  |  |
| q) Waking up during the night because of choking sensation |  |  |  |  |  |  |  |  |  |
| r) Persistent cough |  |  |  |  |  |  |  |  |  |
| s) Stools that float and are difficult to flush |  |  |  |  |  |  |  |  |  |
| t) Diarrhea (>3 times per day) unrelated to eating |  |  |  |  |  |  |  |  |  |
| u) Lack of appetite |  |  |  |  |  |  |  |  |  |
| v) Tiredness |  |  |  |  |  |  |  |  |  |
| w) Low mood |  |  |  |  |  |  |  |  |  |
|  |  |  |  |  |  |  |  |  |  |
| **Symptom** | **Never** | **Rarely** | **Weekly** | **Daily** | **Multiple times per day** |  | **None** | **Some** | **Substantial** |
| x) Reduced energy/activity tolerance |  |  |  |  |  |  |  |  |  |
| y) Voice problems |  |  |  |  |  |  |  |  |  |
| z) Abnormal sensation in fingers and toes |  |  |  |  |  |  |  |  |  |
| aa) Dental problems |  |  |  |  |  |  |  |  |  |
| bb) Hiccups |  |  |  |  |  |  |  |  |  |
| Other (please specify): |  |  |  |  |  |  |  |  |  |
| Other (please specify): |  |  |  |  |  |  |  |  |  |

**Q6. Have you sought medical treatment for any of these symptoms?**

**(Yes or No)**

**Q7. Have you had any of the following medical tests in the last 6 months for the symptoms listed above in Q4?**

| **Test** | **Yes or No** | **Number of times** | | |
| --- | --- | --- | --- | --- |
|  |  | **1** | **2 – 4** | **>5** |
| **Endoscopy (camera test)** |  |  |  |  |
| **CT Scan, X-ray or other radiology** |  |  |  |  |
| **Blood test** |  |  |  |  |
| **Stool test** |  |  |  |  |
| **Other test (specify):** |  |  |  |  |
| **Other test (specify):** |  |  |  |  |

**Q8. Do you take any of the following medications for heartburn and/or reflux symptoms?**

| **Medication** | **Yes or No** | **Frequency** | | | |
| --- | --- | --- | --- | --- | --- |
|  |  | **Daily** | **Weekly** | **Monthly** | **As required** |
| **Proton pump inhibitor (e.g. Omeprazole, lansoprazole)** |  |  |  |  |  |
| **Ranitidine** |  |  |  |  |  |
| **Gaviscon** |  |  |  |  |  |
| **Sucralfate** |  |  |  |  |  |
| **Other medications (specify):** |  |  |  |  |  |

**Q9. Do you take any painkillers or additional medications because of these symptoms?**

| **Medication** | **Yes or No** | **Frequency** | | | |
| --- | --- | --- | --- | --- | --- |
|  |  | **Daily** | **Weekly** | **Monthly** | **As required** |
| **Painkillers** |  |  |  |  |  |
| **Creon** |  |  |  |  |  |
| **Other medications:** |  |  |  |  |  |
|  |  |  |  |  |  |
|  |  |  |  |  |  |

| **Question** | **Answer** |
| --- | --- |
| Q10. Are you continuing to lose weight?  (Yes or No) |  |
| Q11. Do you struggle to keep your weight on?  (Yes or No) |  |
| Q12a. What is your average weight as an adult before your illness (Kg)?  Q12b. What is your current weight (Kg)? |  |
| Q13. What is your current height (cm)? |  |
| Q14. Are your diet and eating habits different from before you were diagnosed with cancer?  (Yes or No) |  |
| Q15. Has this affected your social life?  (Yes or No) |  |
| Q16. How many times a day do you have meals or snacks?  (3 or 4–5 or 6–7 or 8–9) |  |
| Q17. Do you take any supplemental nutrition?  0 – Not at all  1 – Oral  2 – Feeding jejunostomy  3 – By a different route |  |
| Q18. Did you work before you were diagnosed with cancer?  (Yes or No) |  |
| Q19. Have you returned to work?  0 – Now retired  1 – No, I have not returned to work because of my symptoms  2 – Yes, but with some limitations/ reductions in activities  3 – Yes with the same activities as before |  |
| Q20. Are your hobbies and social activities the same as before you were diagnosed with cancer?  (Yes or No) |  |
| Q21. Are you happy that you have survived your cancer?  (Yes or No or I do not wish to answer) |  |
| Please feel free to add free to expand your answer for Q21 here: | |
| Q22. Overall, are you satisfied with your cancer treatment?  (Yes or No) |  |

**Q23. How would you grade your functional status over the past 6 months? (please tick)**

- Fully active able to carry on all pre-disease performance without restriction.
- Restricted in physically strenuous activity but able to carry out work of a light or sedentary nature e.g. light housework, office work.
- Capable of all self-care but unable to carry out any work activities. Up to and about more than 50% of waking hours.
- Capable of only limited self-care, confined to bed or chair more than 50% of waking hours.
- Cannot carry out any self-care. Totally confined to bed or chair.
