# Supplementary material for: Patient-reported outcomes after oesophagectomy in the multicentre LASER study
Source: Br J Surg. 2021 May 11;108(9):1090–6. doi: 10.1093/bjs/znab124 (PMC10364861; doi:10.1093/bjs/znab124)
Supplement: znab124_Supplementary_Data [file znab124_supplementary_data.zip › Appendix S2 - clinical details.pdf]

English

Default Question Block

Patient study identifier

Name of place of surgery

Current age of patient

Gender

- Male
- Female

Height at surgery (cm)

Weight at surgery (kg)

Date of Surgery

Anastomosis location

- Cervical
- Thoracic

Surgical Technique

- Transhiatal
- Ivor-Lewis (2-stage)
- McKeown (3-stage)
- Left thoracoabdominal
- Colonic interposition
- Other - please specify

How was the anastomosis constructed?

- Linear Stapler
- Circular Stapler (please state diameter if known)
- Hand sewn

Alignment of anastomosis

- End to end
- End to side
- Side to side
- Other (Please specify)

Surgical access

- Total Minimally Invasive Esophagectomy
- Hybrid Minimally Invasive Esophagectomy (Open abdominal and thoracoscopy)
- Hybrid Minimally Invasive Esophagectomy (Laparoscopy and open thoracotomy)
- Total Open Esophagectomy

Pyloric management

- No drainage procedure
- Pyloroplasty
- Pyloromyotomy
- Manual pyloric dilatation or stretch
- Botulinum injection
- Other (please specify)
- Unknown

Was a feeding jejunostomy inserted?

- Yes
- No

Neoadjuvant therapy

- Combination chemoradiotherapy
- Radiotherapy alone
- Chemotherapy alone
- None

Chemotherapy or chemoradiotherapy regimen

- CROSS
- MAGIC
- Other
- Unknown

CROSS - Paclitaxel (50 mg/m2) and Carboplatin (Area-Under-Curve = 2) administered by i.v. infusion on days 1, 8, 15, 22, and 29. External beam radiation with a total dose of 41.4 Gy given in 23 fractions of 1.8 Gy, 5 fractions a week.

MAGIC - Three preoperative and three postoperative cycles of intravenous epirubicin (50 mg per square meter of body-surface area) and cisplatin (60 mg per square meter) on day 1, and a continuous intravenous infusion of fluorouracil (200 mg per square meter per day) for 21 days.

Adjuvant therapy

- Combination chemoradiotherapy
- Radiotherapy alone
- Chemotherapy alone
- None

Pathological TNM Stage 7th edition

- Stage I
- Stage II
- Stage III
- Stage IV

Postoperative complications (use complications platform from Low DE et al Annals of Surgery 262(2):286-294 2015)

- Pulmonary
- Cardiac
- Gastrointestinal
- Urologic
- Thromboembolic
- OtherNeurologic/psychiatric
- Infection
- Wound/diaphragm
- Other

Please specify pulmonary complication

- Pneumonia
- Pleural effusion requiring additional drainage procedure
- Pneumothorax requiring treatment
- Atelectasis mucous plugging requiring bronchoscopy
- Respiratory failure requiring reintubation
- Acute respiratory distress syndrome (Berlin Definition)
- Acute aspiration
- Tracheobronchial injury
- Chest tube maintenance for air leak for >10 days postoperatively

Please specify cardiac complication

- Cardiac arrest requiring CPR
- Myocardial infarction as defined by the World Health Organisation
- Dysrhythmia atrial requiring treatment
- Dysrhythmia ventricular requiring treatment
- Congestive heart failure requiring treatment
- Pericarditis requiring treatment

Please specify gastrointestinal complication

- Esophagoenteric leak from anastomosis, stapled line, or localised conduit necrosis
- Conduit necrosis/failure
- Ileus defined as small bowel dysfunction preventing or delayed enteral feeding
- Small bowel obstruction
- Feeding J-tube complication
- Pyloromyotomy/pyloroplasty complication
- Clostridium difficile infection
- Gastrointestinal bleeding requiring intervention or transfusion
- Delayed conduit emptying requiring intervention or delayed discharge or requiring maintenance of NG drainage >7 days postoperatively
- Pancreatitis
- Liver dysfunction

Please specify urological complication

- Acute renal insufficiency (defined as doubling of baseline creatinine)
- Acute renal failure requiring dialysis
- Urinary tract infection
- Urinary retention requiring reinsertion of urinary catheter, delaying discharge, or discharge with a urinary catheter

Please specify thromboembolic complication

- Deep vein thrombosis
- Pulmonary embolus
- Stroke (CVA)
- Peripheral thrombophlebitis

Please specify neurologic/psychiatric complication

- Recurrent nerve injury
- Other neurologic injury
- Acute delirium
- Delirium tremens

Please specify infection complication

- Wound infection requiring opening of wound or antibiotics
- Central IV line infection requiring removal or antibiotics
- Intrathoracic/intra-abdominal abscess
- Generalised sepsis
- Other infections requiring antibiotics

Please specify wound/diaphragm complication

- Thoracic wound dehiscence
- Acute abdominal wall dehiscence/hernia
- Acute diaphragmatic hernia

Please specify other complication

- Chyle leak
- Reoperation for reasons other than bleeding, anastomotic leak, or conduit necrosis
- Multiple organ dysfunction syndrome

Late symptoms requiring intervention (>90 days following surgery)

- Stricture
- Delayed emptying (requiring dilatation)
- Other

Maximum Clavien-Dindo classification of post-operative complication (include 30-day complications or in-patient stay - whichever is the longest)

- I
- II
- III
- IV
