# Supplementary material for: Patient-reported outcomes after oesophagectomy in the multicentre LASER study
Source: Br J Surg. 2021 May 11;108(9):1090–6. doi: 10.1093/bjs/znab124 (PMC10364861; doi:10.1093/bjs/znab124)
Supplement: znab124_Supplementary_Data [file znab124_supplementary_data.zip › Appendix S4 - Online only - Multivariate analysis.pdf]

The LOGISTIC Procedure

Model Information	
Data Set	WORK.C30_OG25A
Response Variable	Chest pain
Number of Response Levels	2
Model	generalized logit
Optimization Technique	Newton-Raphson

Analysis of Maximum Likelihood Estimates

Parameter		Chest pain	DF	Estimate	Standard Error
Intercept		0	1	0.4598	1.398
complications	1	0	1	0.873	0.3288
years_since_surgery		0	1	0.1589	0.0988
neoadjuvant_therapy	Yes	0	1	0.6018	0.3708
surgical_access	Hybrid Minimally Invasive Esophagectomy	0	1	0.2075	0.432
surgical_access	Total Minimally Invasive Esophagectomy	0	1	0.9653	0.4609
surgical_technique	Ivor-Lewis	0	1	-1.1587	0.6485
surgical_technique	Left thoracoabdominal	0	1	-1.848	0.7496
surgical_technique	McKeown (3-stage)	0	1	-1.0459	0.6964
ngender	Female	0	1	-0.9287	0.3415
age_yrs		0	1	0.0271	0.0169

Odds Ratio Estimates

Effect	Chest pain	Point Estimate	95% Wald Confidence Limits	
complications 1 vs 0	0	2.394	1.257	4.56
years_since_surgery	0	1.172	0.966	1.423

neoadjuvant_therapy Yes vs No	0	1.825	0.883	3.775
surgical_access Hybrid Minimally Invasive Esophagectomy vs Total Open Esophagectomy	0	1.231	0.528	2.87
surgical_access Total Minimally Invasive Esophagectomy vs Total Open Esophagectomy	0	2.625	1.064	6.479
surgical_technique Ivor-Lewis vs Transhiatal	0	0.314	0.088	1.119
surgical_technique Left thoracoabdominal vs Transhiatal	0	0.158	0.036	0.685
surgical_technique McKeown (3-stage) vs Transhiatal	0	0.351	0.09	1.376
ngender Female vs Male	0	0.395	0.202	0.771
age_yrs	0	1.028	0.994	1.062

The LOGISTIC Procedure

Model Information	
Data Set	WORK.C30_OG25A
Response Variable	Abdominal pain
Number of Response Levels	2
Model	generalized logit
Optimization Technique	Newton-Raphson

Analysis of Maximum Likelihood Estimates

Parameter	Abdominal pain	DF	Estimate	Standard Error

Intercept		0	1	-1.9492	0.9629
complications	1	0	1	0.1657	0.228
years_since_surgery		0	1	0.1737	0.071
neoadjuvant_therapy	Yes	0	1	0.4035	0.2628
surgical_access	Hybrid Minimally Invasive Esophagectomy	0	1	0.5207	0.3386
surgical_access	Total Minimally Invasive Esophagectomy	0	1	0.3246	0.2706
surgical_technique	Ivor-Lewis	0	1	0.0927	0.3381
surgical_technique	Left thoracoabdominal	0	1	0.0312	0.516
surgical_technique	McKeown (3-stage)	0	1	-0.2696	0.3656
ngender	Female	0	1	-0.272	0.2563
age_yrs		0	1	0.0395	0.0118

Odds Ratio Estimates

Effect	Abdominal pain	Point Estimate	95% Wald Confidence Limits	
complications 1 vs 0	0	1.18	0.755	1.845
years_since_surgery	0	1.19	1.035	1.367
neoadjuvant_therapy Yes vs No	0	1.497	0.894	2.506
surgical_access Hybrid Minimally Invasive Esophagectomy vs Total Open Esophagectomy	0	1.683	0.867	3.269
surgical_access Total Minimally Invasive Esophagectomy vs Total Open Esophagectomy	0	1.384	0.814	2.351
surgical_technique Ivor-Lewis vs Transhiatal	0	1.097	0.566	2.128
surgical_technique Left thoracoabdominal vs Transhiatal	0	1.032	0.375	2.836
surgical_technique McKeown (3-stage) vs Transhiatal	0	0.764	0.373	1.564

**ngender Female vs Male**	**0**	0.762	0.461	1.259
**age_yrs**	**0**	1.04	1.016	1.065

The LOGISTIC Procedure

Model Information	
**Data Set**	WORK.C30_OG25A
**Response Variable**	Pain from scars on chest
**Number of Response Levels**	2
**Model**	generalized logit
**Optimization Technique**	Newton-Raphson

Analysis of Maximum Likelihood Estimates

Parameter		Pain from scars on chest	DF	Estimate	Standard Error
**Intercept**		**0**	1	1.5704	1.5498
**complications**	**1**	**0**	1	-0.3526	0.3642
**years_since_surgery**		**0**	1	0.0938	0.1083
**neoadjuvant_therapy**	**Yes**	**0**	1	-0.7413	0.546
**surgical_access**	**Hybrid Minimally Invasive Esophagectomy**	**0**	1	-0.1372	0.4657
**surgical_access**	**Total Minimally Invasive Esophagectomy**	**0**	1	0.3346	0.458
**surgical_technique**	**Ivor-Lewis**	**0**	1	-0.5077	0.6506
**surgical_technique**	**Left thoracoabdominal**	**0**	1	-0.9553	0.8034
**surgical_technique**	**McKeown (3-stage)**	**0**	1	-0.5297	0.6933
**ngender**	**Female**	**0**	1	-0.4842	0.3886
**age_yrs**		**0**	1	0.0368	0.0184

Effect	Odds Ratio Estimates<br>Pain from scars on chest	Point Estimate	95% Wald<br>Confidence Limits	
complications 1 vs 0	0	0.703	0.344	1.435
years_since_surgery	0	1.098	0.888	1.358
neoadjuvant_therapy Yes vs No	0	0.476	0.163	1.389
surgical_access Hybrid Minimally Invasive Esophagectomy vs Total Open Esophagectomy	0	0.872	0.35	2.172
surgical_access Total Minimally Invasive Esophagectomy vs Total Open Esophagectomy	0	1.397	0.569	3.429
surgical_technique Ivor-Lewis vs Transhiatal	0	0.602	0.168	2.154
surgical_technique Left thoracoabdominal vs Transhiatal	0	0.385	0.08	1.858
surgical_technique McKeown (3-stage) vs Transhiatal	0	0.589	0.151	2.291
ngender Female vs Male	0	0.616	0.288	1.32
age_yrs	0	1.037	1.001	1.076

The LOGISTIC Procedure

Model Information	
Data Set	WORK.C30_OG25A
Response Variable	**Pain from scars on abdomen**
Number of Response Levels	

**Model**	generalized logit
**Optimization Technique**	Newton-Raphson

Analysis of Maximum Likelihood Estimates

Parameter		Pain from scars on abdomen	DF	Estimate	Standard Error
**Intercept**		**0**	1	0.8268	1.9968
**complications**	**1**	**0**	1	-0.203	0.5043
**years_since_surgery**		**0**	1	0.0744	0.1447
**neoadjuvant_therapy**	**Yes**	**0**	1	-0.0157	0.5895
**surgical_access**	**Hybrid Minimally Invasive Esophagectomy**	**0**	1	0.5131	0.8083
**surgical_access**	**Total Minimally Invasive Esophagectomy**	**0**	1	0.5358	0.6114
**surgical_technique**	**Ivor-Lewis**	**0**	1	1.2241	0.6754
**surgical_technique**	**Left thoracoabdominal**	**0**	1	0.2353	0.8818
**surgical_technique**	**McKeown (3-stage)**	**0**	1	0.155	0.64
**ngender**	**Female**	**0**	1	-0.5054	0.5085
**age_yrs**		**0**	1	0.0307	0.0256

Odds Ratio Estimates

Effect	Pain from scars on abdomen	Point Estimate	95% Wald Confidence Limits	
**complications 1 vs 0**	**0**	0.816	0.304	2.193
**years_since_surgery**	**0**	1.077	0.811	1.431
**neoadjuvant_therapy Yes vs No**	**0**	0.984	0.31	3.126
**surgical_access Hybrid Minimally Invasive Esophagectomy vs Total Open Esophagectomy**	**0**	1.67	0.343	8.145
**surgical_access Total Minimally Invasive Esophagectomy vs Total Open Esophagectomy**	**0**	1.709	0.516	5.664

**surgical_technique Ivor-Lewis vs Transhiatal**	**0**	3.401	0.905	12.78
**surgical_technique Left thoracoabdominal vs Transhiatal**	**0**	1.265	0.225	7.125
**surgical_technique McKeown (3-stage) vs Transhiatal**	**0**	1.168	0.333	4.093
**ngender Female vs Male**	**0**	0.603	0.223	1.634
**age_yrs**	**0**	1.031	0.981	1.084

The LOGISTIC Procedure

**Model Information**	
**Data Set**	WORK.C30_OG25A
**Response Variable**	**Difficulty getting food down**
**Number of Response Levels**	
**Model**	generalized logit
**Optimization Technique**	Newton-Raphson

Analysis of Maximum Likelihood Estimates

**Parameter**		**Difficulty getting food**	**DF**	**Estimate**	**Standard Error**
**Intercept**		**0**	1	-1.8127	1.0499
**complications**	**1**	**0**	1	-0.0928	0.2486
**years_since_surgery**		**0**	1	0.2435	0.0786
**neoadjuvant_therapy**	**Yes**	**0**	1	0.1766	0.2903
**surgical_access**	**Hybrid Minimally Invasive Esophagectomy**	**0**	1	0.4166	0.3902

surgical_access	Total Minimally Invasive Esophagectomy	0	1	-0.0665	0.2837
surgical_technique	Ivor-Lewis	0	1	1.1893	0.3145
surgical_technique	Left thoracoabdominal	0	1	0.8534	0.5179
surgical_technique	McKeown (3-stage)	0	1	1.2795	0.3713
ngender	Female	0	1	-0.3592	0.2766
age_yrs		0	1	0.0281	0.0129

Odds Ratio Estimates

Effect	Difficulty getting food down	Point Estimate	95% Wald Confidence Limits	
complications 1 vs 0	0	0.911	0.56	1.484
years_since_surgery	0	1.276	1.094	1.488
neoadjuvant_therapy Yes vs No	0	1.193	0.675	2.108
surgical_access Hybrid Minimally Invasive Esophagectomy vs Total Open Esophagectomy	0	1.517	0.706	3.259
surgical_access Total Minimally Invasive Esophagectomy vs Total Open Esophagectomy	0	0.936	0.537	1.631
surgical_technique Ivor-Lewis vs Transhiatal	0	3.285	1.773	6.084
surgical_technique Left thoracoabdominal vs Transhiatal	0	2.348	0.851	6.479
surgical_technique McKeown (3-stage) vs Transhiatal	0	3.595	1.736	7.443
ngender Female vs Male	0	0.698	0.406	1.201
age_yrs	0	1.028	1.003	1.055

The LOGISTIC Procedure

Model Information	
**Data Set**	WORK.C30_OG25A
**Response Variable**	**Difficulty getting liquids down**
**Number of Response Levels**	
**Model**	generalized logit
**Optimization Technique**	Newton-Raphson

Analysis of Maximum Likelihood Estimates

Parameter		Difficulty getting liquids	DF	Estimate	Standard Error
**Intercept**		**0**	1	0.8738	1.5348
**complications**	**1**	**0**	1	0.238	0.3491
**years_since_surgery**		**0**	1	0.177	0.1073
**neoadjuvant_therapy**	**Yes**	**0**	1	-0.0987	0.4483
**surgical_access**	**Hybrid Minimally Invasive Esophagectomy**	**0**	1	0.7355	0.6572
**surgical_access**	**Total Minimally Invasive Esophagectomy**	**0**	1	-0.0699	0.415
**surgical_technique**	**Ivor-Lewis**	**0**	1	0.807	0.4633
**surgical_technique**	**Left thoracoabdominal**	**0**	1	-0.592	0.5692
**surgical_technique**	**McKeown (3-stage)**	**0**	1	0.8273	0.547
**ngender**	**Female**	**0**	1	-0.4922	0.3792
**age_yrs**		**0**	1	0.0113	0.0191

Odds Ratio Estimates

Effect	Difficulty getting liquids down	Point Estimate	95% Wald Confidence Limits	
**complications 1 vs 0**	**0**	1.269	0.64	2.515
**years_since_surgery**	**0**	1.194	0.967	1.473

**neoadjuvant_therapy Yes vs No**	**0**	0.906	0.376	2.181
**surgical_access Hybrid Minimally Invasive Esophagectomy vs Total Open Esophagectomy**	**0**	2.087	0.575	7.565
**surgical_access Total Minimally Invasive Esophagectomy vs Total Open Esophagectomy**	**0**	0.932	0.413	2.103
**surgical_technique Ivor-Lewis vs Transhiatal**	**0**	2.241	0.904	5.557
**surgical_technique Left thoracoabdominal vs Transhiatal**	**0**	0.553	0.181	1.688
**surgical_technique McKeown (3-stage) vs Transhiatal**	**0**	2.287	0.783	6.682
**ngender Female vs Male**	**0**	0.611	0.291	1.285
**age_yrs**	**0**	1.011	0.974	1.05

The LOGISTIC Procedure

Model Information	
**Data Set**	WORK.C30_OG25A
**Response Variable**	**Regurgitation of food**
**Number of Response Levels**	
**Model**	generalized logit
**Optimization Technique**	Newton-Raphson

Analysis of Maximum Likelihood Estimates				
**Parameter**	**Regurgitation of food**	**DF**	**Estimate**	**Standard Error**

Intercept		0	1	-0.3471	1.0745
complications	1	0	1	0.2431	0.2552
years_since_surgery		0	1	0.1123	0.0784
neoadjuvant_therapy	Yes	0	1	0.2764	0.2989
surgical_access	Hybrid Minimally Invasive Esophagectomy	0	1	-0.1976	0.3591
surgical_access	Total Minimally Invasive Esophagectomy	0	1	-0.2176	0.3041
surgical_technique	Ivor-Lewis	0	1	0.2566	0.3659
surgical_technique	Left thoracoabdominal	0	1	-0.1653	0.5525
surgical_technique	McKeown (3-stage)	0	1	0.4123	0.4157
ngender	Female	0	1	-0.5299	0.28
age_yrs		0	1	0.0262	0.0132

Odds Ratio Estimates

Effect	Regurgitation of food	Point Estimate	95% Wald Confidence Limits	
complications 1 vs 0	0	1.275	0.773	2.103
years_since_surgery	0	1.119	0.96	1.305
neoadjuvant_therapy Yes vs No	0	1.318	0.734	2.368
surgical_access Hybrid Minimally Invasive Esophagectomy vs Total Open Esophagectomy	0	0.821	0.406	1.659
surgical_access Total Minimally Invasive Esophagectomy vs Total Open Esophagectomy	0	0.804	0.443	1.46
surgical_technique Ivor-Lewis vs Transhiatal	0	1.293	0.631	2.648
surgical_technique Left thoracoabdominal vs Transhiatal	0	0.848	0.287	2.503
surgical_technique McKeown (3-stage) vs Transhiatal	0	1.51	0.669	3.411

ngender Female vs Male	0	0.589	0.34	1.019
age_yrs	0	1.027	1	1.053

The LOGISTIC Procedure

Model Information	
Data Set	WORK.C30_OG25A
Response Variable	Nausea
Number of Response Levels	
Model	generalized logit
Optimization Technique	Newton-Raphson

Analysis of Maximum Likelihood Estimates

Parameter		Nausea	DF	Estimate	Standard Error
Intercept		0	1	-0.7588	1.1779
complications	1	0	1	-0.3214	0.2918
years_since_surgery		0	1	0.1569	0.0882
neoadjuvant_therapy	Yes	0	1	0.2642	0.3322
surgical_access	Hybrid Minimally Invasive Esophagectomy	0	1	0.4218	0.4357
surgical_access	Total Minimally Invasive Esophagectomy	0	1	0.0266	0.3387
surgical_technique	Ivor-Lewis	0	1	-0.1407	0.437
surgical_technique	Left thoracoabdominal	0	1	-0.7805	0.5778
surgical_technique	McKeown (3-stage)	0	1	0.1293	0.4892
ngender	Female	0	1	-0.6172	0.3087
age_yrs		0	1	0.042	0.0144

Odds Ratio Estimates				
Effect	Nausea	Point Estimate	95% Wald Confidence Limits	
complications 1 vs 0	0	0.725	0.409	1.285
years_since_surgery	0	1.17	0.984	1.391
neoadjuvant_therapy Yes vs No	0	1.302	0.679	2.497
surgical_access Hybrid Minimally Invasive Esophagectomy vs Total Open Esophagectomy	0	1.525	0.649	3.581
surgical_access Total Minimally Invasive Esophagectomy vs Total Open Esophagectomy	0	1.027	0.529	1.994
surgical_technique Ivor-Lewis vs Transhiatal	0	0.869	0.369	2.046
surgical_technique Left thoracoabdominal vs Transhiatal	0	0.458	0.148	1.422
surgical_technique McKeown (3-stage) vs Transhiatal	0	1.138	0.436	2.969
ngender Female vs Male	0	0.539	0.295	0.988
age_yrs	0	1.043	1.014	1.073

The LOGISTIC Procedure

Model Information	
Data Set	WORK.C30_OG25A
Response Variable	Vomiting
Number of Response Levels	

**Model**	generalized logit
**Optimization Technique**	Newton-Raphson

Analysis of Maximum Likelihood Estimates

Parameter		Vomiting	DF	Estimate	Standard Error
Intercept		0	1	-1.5398	1.3527
complications	1	0	1	0.3024	0.3298
years_since_surgery		0	1	0.1932	0.1027
neoadjuvant_therapy	Yes	0	1	-0.1326	0.4234
surgical_access	Hybrid Minimally Invasive Esophagectomy	0	1	-0.9948	0.4853
surgical_access	Total Minimally Invasive Esophagectomy	0	1	-1.0004	0.4103
surgical_technique	Ivor-Lewis	0	1	0.6187	0.5104
surgical_technique	Left thoracoabdominal	0	1	-0.5609	0.7038
surgical_technique	McKeown (3-stage)	0	1	0.2944	0.5291
ngender	Female	0	1	-1.0088	0.3399
age_yrs		0	1	0.0626	0.0166

Odds Ratio Estimates

Effect	Vomiting	Point Estimate	95% Wald Confidence Limits	
complications 1 vs 0	0	1.353	0.709	2.583
years_since_surgery	0	1.213	0.992	1.484
neoadjuvant_therapy Yes vs No	0	0.876	0.382	2.008
surgical_access Hybrid Minimally Invasive Esophagectomy vs Total Open Esophagectomy	0	0.37	0.143	0.957
surgical_access Total Minimally Invasive Esophagectomy vs Total Open Esophagectomy	0	0.368	0.165	0.822

surgical_technique Ivor-Lewis vs Transhiatal	0	1.857	0.683	5.048
surgical_technique Left thoracoabdominal vs Transhiatal	0	0.571	0.144	2.267
surgical_technique McKeown (3-stage) vs Transhiatal	0	1.342	0.476	3.786
ngender Female vs Male	0	0.365	0.187	0.71
age_yrs	0	1.065	1.031	1.1

The LOGISTIC Procedure

Model Information	
Data Set	WORK.C30_OG25A
Response Variable	Early feeling of fullness after eating
Number of Response Levels	
Model	generalized logit
Optimization Technique	Newton-Raphson

Analysis of Maximum Likelihood Estimates

Parameter		Early feeling of fullness after	DF	Estimate	Standard Error
Intercept		0	1	-1.1456	0.7591
complications	1	0	1	-0.239	0.1763
years_since_surgery		0	1	0.075	0.0529
neoadjuvant_therapy	Yes	0	1	0.1711	0.2078
surgical_access	Hybrid Minimally Invasive Esophagectomy	0	1	0.5061	0.2603

surgical_access	Total Minimally Invasive Esophagectomy	0	1	-0.1396	0.2017
surgical_technique	Ivor-Lewis	0	1	0.4281	0.2468
surgical_technique	Left thoracoabdominal	0	1	0.1526	0.39
surgical_technique	McKeown (3-stage)	0	1	0.2737	0.2734
ngender	Female	0	1	-0.3177	0.2008
age_yrs		0	1	0.0218	0.00931

Odds Ratio Estimates

Effect	Early feeling of fullness after eating	Point Estimate	95% Wald Confidence Limits	
complications 1 vs 0	0	0.787	0.557	1.112
years_since_surgery	0	1.078	0.972	1.196
neoadjuvant_therapy Yes vs No	0	1.187	0.79	1.783
surgical_access Hybrid Minimally Invasive Esophagectomy vs Total Open Esophagectomy	0	1.659	0.996	2.763
surgical_access Total Minimally Invasive Esophagectomy vs Total Open Esophagectomy	0	0.87	0.586	1.292
surgical_technique Ivor-Lewis vs Transhiatal	0	1.534	0.946	2.489
surgical_technique Left thoracoabdominal vs Transhiatal	0	1.165	0.542	2.502
surgical_technique McKeown (3-stage) vs Transhiatal	0	1.315	0.769	2.247
ngender Female vs Male	0	0.728	0.491	1.079
age_yrs	0	1.022	1.004	1.041

The LOGISTIC Procedure

Model Information	
Data Set	WORK.C30_OG25A
Response Variable	Heart palpitation after eating
Number of Response Levels	
Model	generalized logit
Optimization Technique	Newton-Raphson

Analysis of Maximum Likelihood Estimates					
Parameter		Heart palpitation after	DF	Estimate	Standard Error
Intercept		0	1	-0.9235	1.5059
complications	1	0	1	0.3339	0.3596
years_since_surgery		0	1	-0.0282	0.1064
neoadjuvant_therapy	Yes	0	1	0.00527	0.4576
surgical_access	Hybrid Minimally Invasive Esophagectomy	0	1	0.8742	0.6596
surgical_access	Total Minimally Invasive Esophagectomy	0	1	-0.0755	0.4207
surgical_technique	Ivor-Lewis	0	1	-1.5372	0.799
surgical_technique	Left thoracoabdominal	0	1	-2.3428	0.8942
surgical_technique	McKeown (3-stage)	0	1	-1.4986	0.8263
ngender	Female	0	1	-1.1263	0.3687
age_yrs		0	1	0.0895	0.0181

Odds Ratio Estimates				
Effect	Heart palpitation after eating	Point Estimate	95% Wald Confidence Limits	
complications 1 vs 0	0	1.396	0.69	2.826
years_since_surgery	0	0.972	0.789	1.198

**neoadjuvant_therapy Yes vs No**	**0**	1.005	0.41	2.465
**surgical_access Hybrid Minimally Invasive Esophagectomy vs Total Open Esophagectomy**	**0**	2.397	0.658	8.732
**surgical_access Total Minimally Invasive Esophagectomy vs Total Open Esophagectomy**	**0**	0.927	0.407	2.115
**surgical_technique Ivor-Lewis vs Transhiatal**	**0**	0.215	0.045	1.029
**surgical_technique Left thoracoabdominal vs Transhiatal**	**0**	0.096	0.017	0.554
**surgical_technique McKeown (3-stage) vs Transhiatal**	**0**	0.223	0.044	1.129
**ngender Female vs Male**	**0**	0.324	0.157	0.668
**age_yrs**	**0**	1.094	1.056	1.133

The LOGISTIC Procedure

Model Information	
**Data Set**	WORK.C30_OG25A
**Response Variable**	**Sweating after eating**
**Number of Response Levels**	
**Model**	generalized logit
**Optimization Technique**	Newton-Raphson

Analysis of Maximum Likelihood Estimates				
**Parameter**	**Sweating after eating**	**DF**	**Estimate**	**Standard Error**

Intercept		0	1	-1.2406	1.5269
complications	1	0	1	0.4845	0.375
years_since_surgery		0	1	0.0186	0.1113
neoadjuvant_therapy	Yes	0	1	0.4722	0.4276
surgical_access	Hybrid Minimally Invasive Esophagectomy	0	1	0.716	0.5936
surgical_access	Total Minimally Invasive Esophagectomy	0	1	0.5635	0.4677
surgical_technique	Ivor-Lewis	0	1	-1.6047	0.8128
surgical_technique	Left thoracoabdominal	0	1	-1.914	0.9471
surgical_technique	McKeown (3-stage)	0	1	-1.8157	0.8307
ngender	Female	0	1	-1.479	0.3797
age_yrs		0	1	0.0867	0.0187

Odds Ratio Estimates

Effect	Sweating after eating	Point Estimate	95% Wald Confidence Limits	
complications 1 vs 0	0	1.623	0.779	3.385
years_since_surgery	0	1.019	0.819	1.267
neoadjuvant_therapy Yes vs No	0	1.603	0.694	3.707
surgical_access Hybrid Minimally Invasive Esophagectomy vs Total Open Esophagectomy	0	2.046	0.639	6.55
surgical_access Total Minimally Invasive Esophagectomy vs Total Open Esophagectomy	0	1.757	0.702	4.393
surgical_technique Ivor-Lewis vs Transhiatal	0	0.201	0.041	0.989
surgical_technique Left thoracoabdominal vs Transhiatal	0	0.147	0.023	0.944
surgical_technique McKeown (3-stage) vs Transhiatal	0	0.163	0.032	0.829

ngender Female vs Male	0	0.228	0.108	0.48
age_yrs	0	1.091	1.051	1.131

The LOGISTIC Procedure

Model Information	
Data Set	WORK.C30_OG25A
Response Variable	Dizziness after eating
Number of Response Levels	
Model	generalized logit
Optimization Technique	Newton-Raphson

Analysis of Maximum Likelihood Estimates

Parameter		Dizziness after eating	DF	Estimate	Standard Error
Intercept		0	1	-0.5598	1.4996
complications	1	0	1	0.5142	0.3755
years_since_surgery		0	1	0.0904	0.1121
neoadjuvant_therapy	Yes	0	1	0.6829	0.4089
surgical_access	Hybrid Minimally Invasive Esophagectomy	0	1	0.4154	0.5413
surgical_access	Total Minimally Invasive Esophagectomy	0	1	0.9008	0.5275
surgical_technique	Ivor-Lewis	0	1	-0.0178	0.5194
surgical_technique	Left thoracoabdominal	0	1	-0.4339	0.702
surgical_technique	McKeown (3-stage)	0	1	0.4398	0.6445
ngender	Female	0	1	-0.5639	0.4057
age_yrs		0	1	0.0356	0.0189

Odds Ratio Estimates				
**Effect**	**Dizziness after eating**	**Point Estimate**	**95% Wald Confidence Limits**	
**complications 1 vs 0**	**0**	1.672	0.801	3.491
**years_since_surgery**	**0**	1.095	0.879	1.364
**neoadjuvant_therapy Yes vs No**	**0**	1.98	0.888	4.412
**surgical_access Hybrid Minimally Invasive Esophagectomy vs Total Open Esophagectomy**	**0**	1.515	0.524	4.377
**surgical_access Total Minimally Invasive Esophagectomy vs Total Open Esophagectomy**	**0**	2.461	0.875	6.922
**surgical_technique Ivor-Lewis vs Transhiatal**	**0**	0.982	0.355	2.719
**surgical_technique Left thoracoabdominal vs Transhiatal**	**0**	0.648	0.164	2.565
**surgical_technique McKeown (3-stage) vs Transhiatal**	**0**	1.552	0.439	5.49
**ngender Female vs Male**	**0**	0.569	0.257	1.26
**age_yrs**	**0**	1.036	0.999	1.075

The LOGISTIC Procedure

Model Information	
**Data Set**	WORK.C30_OG25A
**Response Variable**	**Bloating or cramping after eating**
**Number of Response Levels**	

**Model**	generalized logit
**Optimization Technique**	Newton-Raphson

Analysis of Maximum Likelihood Estimates

Parameter		Bloating or cramping after	DF	Estimate	Standard Error
Intercept		0	1	-1.7839	0.9706
complications	1	0	1	-0.2393	0.24
years_since_surgery		0	1	0.0383	0.07
neoadjuvant_therapy	Yes	0	1	0.4019	0.2682
surgical_access	Hybrid Minimally Invasive Esophagectomy	0	1	0.374	0.3607
surgical_access	Total Minimally Invasive Esophagectomy	0	1	0.0103	0.2739
surgical_technique	Ivor-Lewis	0	1	0.273	0.3435
surgical_technique	Left thoracoabdominal	0	1	-0.3868	0.4884
surgical_technique	McKeown (3-stage)	0	1	-0.1332	0.3621
ngender	Female	0	1	-0.5811	0.2531
age_yrs		0	1	0.0528	0.0121

Odds Ratio Estimates

Effect	Bloating or cramping after eating	Point Estimate	95% Wald Confidence Limits	
complications 1 vs 0	0	0.787	0.492	1.26
years_since_surgery	0	1.039	0.906	1.192
neoadjuvant_therapy Yes vs No	0	1.495	0.884	2.529
surgical_access Hybrid Minimally Invasive Esophagectomy vs Total Open Esophagectomy	0	1.454	0.717	2.948
surgical_access Total Minimally Invasive Esophagectomy vs Total Open Esophagectomy	0	1.01	0.591	1.728

**surgical_technique Ivor-Lewis vs Transhiatal**	**0**	1.314	0.67	2.576
**surgical_technique Left thoracoabdominal vs Transhiatal**	**0**	0.679	0.261	1.769
**surgical_technique McKeown (3-stage) vs Transhiatal**	**0**	0.875	0.43	1.78
**ngender Female vs Male**	**0**	0.559	0.341	0.919
**age_yrs**	**0**	1.054	1.03	1.079

The LOGISTIC Procedure

**Model Information**	
**Data Set**	WORK.C30_OG25A
**Response Variable**	**Loose bowel motions / diarrhea after eating**
**Number of Response Levels**	
**Model**	generalized logit
**Optimization Technique**	Newton-Raphson

Analysis of Maximum Likelihood Estimates

**Parameter**		**Loose bowel motions /**	**DF**	**Estimate**	**Standard Error**
**Intercept**		**0**	1	-0.3149	1.0574
**complications**	**1**	**0**	1	-0.0759	0.261
**years_since_surgery**		**0**	1	-0.0374	0.0773
**neoadjuvant_therapy**	**Yes**	**0**	1	0.1477	0.3065
**surgical_access**	**Hybrid Minimally Invasive Esophagectomy**	**0**	1	0.7295	0.3969

surgical_access	Total Minimally Invasive Esophagectomy	0	1	0.6588	0.3344
surgical_technique	Ivor-Lewis	0	1	-0.4295	0.3801
surgical_technique	Left thoracoabdominal	0	1	-0.1814	0.5642
surgical_technique	McKeown (3-stage)	0	1	0.2373	0.4524
ngender	Female	0	1	-0.66	0.2845
age_yrs		0	1	0.0421	0.0132

Odds Ratio Estimates

Effect	Loose bowel motions / diarrhea after eating	Point Estimate	95% Wald Confidence Limits	
complications 1 vs 0	0	0.927	0.556	1.546
years_since_surgery	0	0.963	0.828	1.121
neoadjuvant_therapy Yes vs No	0	1.159	0.636	2.114
surgical_access Hybrid Minimally Invasive Esophagectomy vs Total Open Esophagectomy	0	2.074	0.953	4.515
surgical_access Total Minimally Invasive Esophagectomy vs Total Open Esophagectomy	0	1.933	1.004	3.722
surgical_technique Ivor-Lewis vs Transhiatal	0	0.651	0.309	1.371
surgical_technique Left thoracoabdominal vs Transhiatal	0	0.834	0.276	2.521
surgical_technique McKeown (3-stage) vs Transhiatal	0	1.268	0.522	3.077
ngender Female vs Male	0	0.517	0.296	0.903
age_yrs	0	1.043	1.016	1.07

The LOGISTIC Procedure

Model Information	
Data Set	WORK.C30_OG25A
Response Variable	Heartburn/acid/bile regurgitation
Number of Response Levels	
Model	generalized logit
Optimization Technique	Newton-Raphson

Analysis of Maximum Likelihood Estimates					
Parameter		Heartburn/acid/bile	DF	Estimate	Standard Error
Intercept		0	1	-0.5592	0.874
complications	1	0	1	0.1927	0.2049
years_since_surgery		0	1	-0.00557	0.0614
neoadjuvant_therapy	Yes	0	1	0.1659	0.2455
surgical_access	Hybrid Minimally Invasive Esophagectomy	0	1	0.0272	0.3028
surgical_access	Total Minimally Invasive Esophagectomy	0	1	-0.4131	0.2388
surgical_technique	Ivor-Lewis	0	1	0.052	0.3038
surgical_technique	Left thoracoabdominal	0	1	-0.6064	0.4417
surgical_technique	McKeown (3-stage)	0	1	0.0437	0.3334
ngender	Female	0	1	-0.5057	0.2278
age_yrs		0	1	0.0329	0.0107

Odds Ratio Estimates				
Effect	Heartburn/acid/bile regurgitation	Point Estimate	95% Wald Confidence Limits	
complications 1 vs 0	0	1.213	0.812	1.812
years_since_surgery	0	0.994	0.882	1.122

**neoadjuvant_therapy Yes vs No**	**0**	1.18	0.73	1.91
**surgical_access Hybrid Minimally Invasive Esophagectomy vs Total Open Esophagectomy**	**0**	1.028	0.568	1.86
**surgical_access Total Minimally Invasive Esophagectomy vs Total Open Esophagectomy**	**0**	0.662	0.414	1.056
**surgical_technique Ivor-Lewis vs Transhiatal**	**0**	1.053	0.581	1.911
**surgical_technique Left thoracoabdominal vs Transhiatal**	**0**	0.545	0.229	1.296
**surgical_technique McKeown (3-stage) vs Transhiatal**	**0**	1.045	0.544	2.008
**ngender Female vs Male**	**0**	0.603	0.386	0.942
**age_yrs**	**0**	1.033	1.012	1.055

The LOGISTIC Procedure

Model Information	
**Data Set**	WORK.C30_OG25A
**Response Variable**	**Waking up during the night because of choking sensation**
**Number of Response Levels**	
**Model**	generalized logit
**Optimization Technique**	Newton-Raphson

Analysis of Maximum Likelihood Estimates

Parameter	Waking up during the night	DF	Estimate	Standard Error

Intercept		0	1	-0.9348	1.4568
complications	1	0	1	0.2972	0.3687
years_since_surgery		0	1	0.05	0.1109
neoadjuvant_therapy	Yes	0	1	0.4212	0.4174
surgical_access	Hybrid Minimally Invasive Esophagectomy	0	1	-0.2227	0.5024
surgical_access	Total Minimally Invasive Esophagectomy	0	1	0.0205	0.4542
surgical_technique	Ivor-Lewis	0	1	-0.394	0.6045
surgical_technique	Left thoracoabdominal	0	1	-0.7193	0.8216
surgical_technique	McKeown (3-stage)	0	1	-0.2349	0.6584
ngender	Female	0	1	-0.8495	0.3848
age_yrs		0	1	0.0619	0.0179

Odds Ratio Estimates

Effect	Waking up during the night because of choking sensation	Point Estimate	95% Wald Confidence Limits	
complications 1 vs 0	0	1.346	0.654	2.773
years_since_surgery	0	1.051	0.846	1.307
neoadjuvant_therapy Yes vs No	0	1.524	0.672	3.453
surgical_access Hybrid Minimally Invasive Esophagectomy vs Total Open Esophagectomy	0	0.8	0.299	2.143
surgical_access Total Minimally Invasive Esophagectomy vs Total Open Esophagectomy	0	1.021	0.419	2.486
surgical_technique Ivor-Lewis vs Transhiatal	0	0.674	0.206	2.205
surgical_technique Left thoracoabdominal vs Transhiatal	0	0.487	0.097	2.437
surgical_technique McKeown (3-stage) vs Transhiatal	0	0.791	0.218	2.873

ngender Female vs Male	0	0.428	0.201	0.909
age_yrs	0	1.064	1.027	1.102

The LOGISTIC Procedure

Model Information	
Data Set	WORK.C30_OG25A
Response Variable	Persistent cough
Number of Response Levels	
Model	generalized logit
Optimization Technique	Newton-Raphson

Analysis of Maximum Likelihood Estimates

Parameter		Persistent cough	DF	Estimate	Standard Error
Intercept		0	1	2.1419	1.0236
complications	1	0	1	0.0436	0.234
years_since_surgery		0	1	-0.018	0.0701
neoadjuvant_therapy	Yes	0	1	0.4029	0.2632
surgical_access	Hybrid Minimally Invasive Esophagectomy	0	1	-0.1746	0.3243
surgical_access	Total Minimally Invasive Esophagectomy	0	1	-0.5055	0.2671
surgical_technique	Ivor-Lewis	0	1	-0.3574	0.3734
surgical_technique	Left thoracoabdominal	0	1	-0.1515	0.6329
surgical_technique	McKeown (3-stage)	0	1	-0.5232	0.3946
ngender	Female	0	1	-0.3926	0.2576
age_yrs		0	1	0.00166	0.0123

Effect	Persistent cough	Point Estimate	95% Wald Confidence Limits	
complications 1 vs 0	0	1.045	0.66	1.652
years_since_surgery	0	0.982	0.856	1.127
neoadjuvant_therapy Yes vs No	0	1.496	0.893	2.506
surgical_access Hybrid Minimally Invasive Esophagectomy vs Total Open Esophagectomy	0	0.84	0.445	1.586
surgical_access Total Minimally Invasive Esophagectomy vs Total Open Esophagectomy	0	0.603	0.357	1.018
surgical_technique Ivor-Lewis vs Transhiatal	0	0.699	0.336	1.454
surgical_technique Left thoracoabdominal vs Transhiatal	0	0.859	0.249	2.971
surgical_technique McKeown (3-stage) vs Transhiatal	0	0.593	0.273	1.284
ngender Female vs Male	0	0.675	0.408	1.119
age_yrs	0	1.002	0.978	1.026

Odds Ratio Estimates

The LOGISTIC Procedure

Model Information

Data Set	WORK.C30_OG25A
Response Variable	Stools that float and are difficult to flush
Number of Response Levels	

**Model**	generalized logit
**Optimization Technique**	Newton-Raphson

Analysis of Maximum Likelihood Estimates

Parameter		Stools that float and are difficult	DF	Estimate	Standard Error
Intercept		0	1	-0.00526	1.5124
complications	1	0	1	0.5164	0.3889
years_since_surgery		0	1	-0.0619	0.1155
neoadjuvant_therapy	Yes	0	1	0.738	0.4057
surgical_access	Hybrid Minimally Invasive Esophagectomy	0	1	0.0261	0.5072
surgical_access	Total Minimally Invasive Esophagectomy	0	1	0.9266	0.5252
surgical_technique	Ivor-Lewis	0	1	0.5524	0.4955
surgical_technique	Left thoracoabdominal	0	1	13.4945	446.3
surgical_technique	McKeown (3-stage)	0	1	0.0955	0.5366
ngender	Female	0	1	-0.4437	0.4143
age_yrs		0	1	0.033	0.0192

Odds Ratio Estimates

Effect	Stools that float and are difficult to flush	Point Estimate	95% Wald Confidence Limits	
complications 1 vs 0	0	1.676	0.782	3.592
years_since_surgery	0	0.94	0.75	1.179
neoadjuvant_therapy Yes vs No	0	2.092	0.944	4.633
surgical_access Hybrid Minimally Invasive Esophagectomy vs Total Open Esophagectomy	0	1.026	0.38	2.774
surgical_access Total Minimally Invasive Esophagectomy vs Total Open Esophagectomy	0	2.526	0.902	7.071

**surgical_technique Ivor-Lewis vs Transhiatal**	**0**	1.737	0.658	4.589
**surgical_technique Left thoracoabdominal vs Transhiatal**	**0**	>999.999	<0.001	>999.999
**surgical_technique McKeown (3-stage) vs Transhiatal**	**0**	1.1	0.384	3.149
**ngender Female vs Male**	**0**	0.642	0.285	1.445
**age_yrs**	**0**	1.034	0.995	1.073

The LOGISTIC Procedure

**Model Information**	
**Data Set**	WORK.C30_OG25A
**Response Variable**	**Diarrhea unrelated to eating**
**Number of Response Levels**	
**Model**	generalized logit
**Optimization Technique**	Newton-Raphson

Analysis of Maximum Likelihood Estimates

**Parameter**		**Diarrhea unrelated to**	**DF**	**Estimate**	**Standard Error**
**Intercept**		**0**	1	0.2159	1.5399
**complications**	**1**	**0**	1	-0.0128	0.3887
**years_since_surgery**		**0**	1	-0.0384	0.1123
**neoadjuvant_therapy**	**Yes**	**0**	1	0.1871	0.4595
**surgical_access**	**Hybrid Minimally Invasive Esophagectomy**	**0**	1	-0.2234	0.5718

surgical_access	Total Minimally Invasive Esophagectomy	0	1	-0.328	0.4712
surgical_technique	Ivor-Lewis	0	1	-0.1814	0.6168
surgical_technique	Left thoracoabdominal	0	1	-1.3735	0.7439
surgical_technique	McKeown (3-stage)	0	1	0.019	0.6725
ngender	Female	0	1	-0.878	0.4002
age_yrs		0	1	0.0568	0.0188

Odds Ratio Estimates

Effect	Diarrhea unrelated to eating	Point Estimate	95% Wald Confidence Limits	
complications 1 vs 0	0	0.987	0.461	2.115
years_since_surgery	0	0.962	0.772	1.199
neoadjuvant_therapy Yes vs No	0	1.206	0.49	2.968
surgical_access Hybrid Minimally Invasive Esophagectomy vs Total Open Esophagectomy	0	0.8	0.261	2.453
surgical_access Total Minimally Invasive Esophagectomy vs Total Open Esophagectomy	0	0.72	0.286	1.814
surgical_technique Ivor-Lewis vs Transhiatal	0	0.834	0.249	2.794
surgical_technique Left thoracoabdominal vs Transhiatal	0	0.253	0.059	1.088
surgical_technique McKeown (3-stage) vs Transhiatal	0	1.019	0.273	3.808
ngender Female vs Male	0	0.416	0.19	0.911
age_yrs	0	1.058	1.02	1.098

The LOGISTIC Procedure

Model Information	
Data Set	WORK.C30_OG25A
Response Variable	Lack of appetite
Number of Response Levels	
Model	generalized logit
Optimization Technique	Newton-Raphson

Analysis of Maximum Likelihood Estimates

Parameter		Lack of appetite	DF	Estimate	Standard Error
Intercept		0	1	1.3014	1.0924
complications	1	0	1	-0.2785	0.2631
years_since_surgery		0	1	0.0337	0.0765
neoadjuvant_therapy	Yes	0	1	0.4061	0.2819
surgical_access	Hybrid Minimally Invasive Esophagectomy	0	1	0.246	0.3722
surgical_access	Total Minimally Invasive Esophagectomy	0	1	0.0868	0.294
surgical_technique	Ivor-Lewis	0	1	0.6816	0.3368
surgical_technique	Left thoracoabdominal	0	1	0.9652	0.6656
surgical_technique	McKeown (3-stage)	0	1	0.289	0.3613
ngender	Female	0	1	-0.3746	0.2793
age_yrs		0	1	0.00116	0.0136

Odds Ratio Estimates

Effect	Lack of appetite	Point Estimate	95% Wald Confidence Limits	
complications 1 vs 0	0	0.757	0.452	1.268
years_since_surgery	0	1.034	0.89	1.202

**neoadjuvant_therapy Yes vs No**	**0**	1.501	0.864	2.608
**surgical_access Hybrid Minimally Invasive Esophagectomy vs Total Open Esophagectomy**	**0**	1.279	0.617	2.652
**surgical_access Total Minimally Invasive Esophagectomy vs Total Open Esophagectomy**	**0**	1.091	0.613	1.941
**surgical_technique Ivor-Lewis vs Transhiatal**	**0**	1.977	1.022	3.826
**surgical_technique Left thoracoabdominal vs Transhiatal**	**0**	2.625	0.712	9.678
**surgical_technique McKeown (3-stage) vs Transhiatal**	**0**	1.335	0.658	2.71
**ngender Female vs Male**	**0**	0.688	0.398	1.189
**age_yrs**	**0**	1.001	0.975	1.028

The LOGISTIC Procedure

**Model Information**	
**Data Set**	WORK.C30_OG25A
**Response Variable**	**Tiredness**
**Number of Response Levels**	
**Model**	generalized logit
**Optimization Technique**	Newton-Raphson

**Analysis of Maximum Likelihood Estimates**				
**Parameter**	**Tiredness**	**DF**	**Estimate**	**Standard Error**

Intercept		0	1	-1.5325	0.7679
complications	1	0	1	-0.065	0.1783
years_since_surgery		0	1	0.0308	0.0535
neoadjuvant_therapy	Yes	0	1	0.4836	0.206
surgical_access	Hybrid Minimally Invasive Esophagectomy	0	1	0.1342	0.2501
surgical_access	Total Minimally Invasive Esophagectomy	0	1	-0.1368	0.2074
surgical_technique	Ivor-Lewis	0	1	0.0194	0.2587
surgical_technique	Left thoracoabdominal	0	1	0.1546	0.4232
surgical_technique	McKeown (3-stage)	0	1	0.00331	0.2861
ngender	Female	0	1	-0.2572	0.205
age_yrs		0	1	0.0321	0.00941

Odds Ratio Estimates

Effect	Tiredness	Point Estimate	95% Wald Confidence Limits	
complications 1 vs 0	0	0.937	0.661	1.329
years_since_surgery	0	1.031	0.929	1.145
neoadjuvant_therapy Yes vs No	0	1.622	1.083	2.429
surgical_access Hybrid Minimally Invasive Esophagectomy vs Total Open Esophagectomy	0	1.144	0.7	1.867
surgical_access Total Minimally Invasive Esophagectomy vs Total Open Esophagectomy	0	0.872	0.581	1.309
surgical_technique Ivor-Lewis vs Transhiatal	0	1.02	0.614	1.693
surgical_technique Left thoracoabdominal vs Transhiatal	0	1.167	0.509	2.676
surgical_technique McKeown (3-stage) vs Transhiatal	0	1.003	0.573	1.758

**ngender Female vs Male**	**0**		0.773	0.517	1.156
**age_yrs**	**0**		1.033	1.014	1.052

The LOGISTIC Procedure

Model Information	
**Data Set**	WORK.C30_OG25A
**Response Variable**	**Low mood**
**Number of Response Levels**	
**Model**	generalized logit
**Optimization Technique**	Newton-Raphson

Analysis of Maximum Likelihood Estimates

Parameter		Low mood	DF	Estimate	Standard Error
**Intercept**		**0**	1	-0.6848	1.0902
**complications**	**1**	**0**	1	0.1741	0.2622
**years_since_surgery**		**0**	1	0.0805	0.0796
**neoadjuvant_therapy**	**Yes**	**0**	1	0.4733	0.2918
**surgical_access**	**Hybrid Minimally Invasive Esophagectomy**	**0**	1	0.4971	0.4011
**surgical_access**	**Total Minimally Invasive Esophagectomy**	**0**	1	0.2669	0.3077
**surgical_technique**	**Ivor-Lewis**	**0**	1	0.5396	0.3469
**surgical_technique**	**Left thoracoabdominal**	**0**	1	0.6475	0.6062
**surgical_technique**	**McKeown (3-stage)**	**0**	1	0.309	0.3875
**ngender**	**Female**	**0**	1	-0.1519	0.2975
**age_yrs**		**0**	1	0.0243	0.0136

Effect	Odds Ratio Estimates<br>Low mood	Point Estimate	95% Wald Confidence Limits	
complications 1 vs 0	0	1.19	0.712	1.99
years_since_surgery	0	1.084	0.927	1.267
neoadjuvant_therapy Yes vs No	0	1.605	0.906	2.844
surgical_access Hybrid Minimally Invasive Esophagectomy vs Total Open Esophagectomy	0	1.644	0.749	3.609
surgical_access Total Minimally Invasive Esophagectomy vs Total Open Esophagectomy	0	1.306	0.715	2.387
surgical_technique Ivor-Lewis vs Transhiatal	0	1.715	0.869	3.386
surgical_technique Left thoracoabdominal vs Transhiatal	0	1.911	0.582	6.269
surgical_technique McKeown (3-stage) vs Transhiatal	0	1.362	0.637	2.911
ngender Female vs Male	0	0.859	0.48	1.539
age_yrs	0	1.025	0.998	1.052

The LOGISTIC Procedure

Model Information	
Data Set	WORK.C30_OG25A
Response Variable	Reduced energy or activity tolerance
Number of Response Levels	

**Model**	generalized logit
**Optimization Technique**	Newton-Raphson

Analysis of Maximum Likelihood Estimates

Parameter		Reduced energy or activity	DF	Estimate	Standard Error
Intercept		0	1	0.0713	0.7558
complications	1	0	1	-0.212	0.1743
years_since_surgery		0	1	-0.0108	0.0521
neoadjuvant_therapy	Yes	0	1	0.0366	0.2081
surgical_access	Hybrid Minimally Invasive Esophagectomy	0	1	0.1237	0.2486
surgical_access	Total Minimally Invasive Esophagectomy	0	1	-0.4646	0.2002
surgical_technique	Ivor-Lewis	0	1	0.3999	0.2465
surgical_technique	Left thoracoabdominal	0	1	0.2016	0.3995
surgical_technique	McKeown (3-stage)	0	1	0.1565	0.2714
ngender	Female	0	1	0.0701	0.2052
age_yrs		0	1	0.0114	0.00925

Odds Ratio Estimates

Effect	Reduced energy or activity tolerance	Point Estimate	95% Wald Confidence Limits	
complications 1 vs 0	0	0.809	0.575	1.138
years_since_surgery	0	0.989	0.893	1.096
neoadjuvant_therapy Yes vs No	0	1.037	0.69	1.56
surgical_access Hybrid Minimally Invasive Esophagectomy vs Total Open Esophagectomy	0	1.132	0.695	1.842
surgical_access Total Minimally Invasive Esophagectomy vs Total Open Esophagectomy	0	0.628	0.424	0.93

**surgical_technique Ivor-Lewis vs Transhiatal**	**0**	1.492	0.92	2.418
**surgical_technique Left thoracoabdominal vs Transhiatal**	**0**	1.223	0.559	2.677
**surgical_technique McKeown (3-stage) vs Transhiatal**	**0**	1.169	0.687	1.99
**ngender Female vs Male**	**0**	1.073	0.717	1.604
**age_yrs**	**0**	1.011	0.993	1.03

The LOGISTIC Procedure

Model Information	
**Data Set**	WORK.C30_OG25A
**Response Variable**	**Voice problems**
**Number of Response Levels**	
**Model**	generalized logit
**Optimization Technique**	Newton-Raphson

Analysis of Maximum Likelihood Estimates

Parameter		Voice problems	DF	Estimate	Standard Error
**Intercept**		**0**	1	2.1506	1.196
**complications**	**1**	**0**	1	-0.1105	0.2763
**years_since_surgery**		**0**	1	0.0722	0.0834
**neoadjuvant_therapy**	**Yes**	**0**	1	0.1471	0.3187
**surgical_access**	**Hybrid Minimally Invasive Esophagectomy**	**0**	1	-0.4637	0.3499

surgical_access	Total Minimally Invasive Esophagectomy	0	1	-0.1227	0.3257
surgical_technique	Ivor-Lewis	0	1	0.2084	0.3998
surgical_technique	Left thoracoabdominal	0	1	0.7207	0.8106
surgical_technique	McKeown (3-stage)	0	1	-0.0284	0.4294
ngender	Female	0	1	-0.2487	0.3081
age_yrs		0	1	-0.00261	0.0147

Odds Ratio Estimates

Effect	Voice problems	Point Estimate	95% Wald Confidence Limits	
complications 1 vs 0	0	0.895	0.521	1.539
years_since_surgery	0	1.075	0.913	1.266
neoadjuvant_therapy Yes vs No	0	1.158	0.62	2.164
surgical_access Hybrid Minimally Invasive Esophagectomy vs Total Open Esophagectomy	0	0.629	0.317	1.249
surgical_access Total Minimally Invasive Esophagectomy vs Total Open Esophagectomy	0	0.884	0.467	1.675
surgical_technique Ivor-Lewis vs Transhiatal	0	1.232	0.563	2.697
surgical_technique Left thoracoabdominal vs Transhiatal	0	2.056	0.42	10.069
surgical_technique McKeown (3-stage) vs Transhiatal	0	0.972	0.419	2.255
ngender Female vs Male	0	0.78	0.426	1.426
age_yrs	0	0.997	0.969	1.027

The LOGISTIC Procedure

Model Information	
Data Set	WORK.C30_OG25A
Response Variable	Abnormal sensation in fingers and toes
Number of Response Levels	
Model	generalized logit
Optimization Technique	Newton-Raphson

Analysis of Maximum Likelihood Estimates					
Parameter		Abnormal sensation in	DF	Estimate	Standard Error
Intercept		0	1	0.5857	1.0722
complications	1	0	1	0.039	0.2496
years_since_surgery		0	1	0.1358	0.0774
neoadjuvant_therapy	Yes	0	1	0.1687	0.2908
surgical_access	Hybrid Minimally Invasive Esophagectomy	0	1	-0.2507	0.339
surgical_access	Total Minimally Invasive Esophagectomy	0	1	-0.2193	0.2879
surgical_technique	Ivor-Lewis	0	1	0.722	0.3315
surgical_technique	Left thoracoabdominal	0	1	1.4496	0.7813
surgical_technique	McKeown (3-stage)	0	1	0.59	0.3701
ngender	Female	0	1	-0.2375	0.2822
age_yrs		0	1	0.00489	0.0133

Odds Ratio Estimates				
Effect	Abnormal sensation in fingers and toes	Point Estimate	95% Wald Confidence Limits	
complications 1 vs 0	0	1.04	0.637	1.696
years_since_surgery	0	1.145	0.984	1.333

**neoadjuvant_therapy Yes vs No**	**0**	1.184	0.669	2.093
**surgical_access Hybrid Minimally Invasive Esophagectomy vs Total Open Esophagectomy**	**0**	0.778	0.4	1.512
**surgical_access Total Minimally Invasive Esophagectomy vs Total Open Esophagectomy**	**0**	0.803	0.457	1.412
**surgical_technique Ivor-Lewis vs Transhiatal**	**0**	2.059	1.075	3.942
**surgical_technique Left thoracoabdominal vs Transhiatal**	**0**	4.261	0.922	19.703
**surgical_technique McKeown (3-stage) vs Transhiatal**	**0**	1.804	0.873	3.726
**ngender Female vs Male**	**0**	0.789	0.454	1.371
**age_yrs**	**0**	1.005	0.979	1.031

The LOGISTIC Procedure

**Model Information**	
**Data Set**	WORK.C30_OG25A
**Response Variable**	**Dental problems**
**Number of Response Levels**	
**Model**	generalized logit
**Optimization Technique**	Newton-Raphson

Analysis of Maximum Likelihood Estimates

**Parameter**	**Dental problems**	**DF**	**Estimate**	**Standard Error**

Intercept		0	1	1.2456	1.5308
complications	1	0	1	0.0933	0.3771
years_since_surgery		0	1	0.0135	0.1098
neoadjuvant_therapy	Yes	0	1	0.3592	0.4184
surgical_access	Hybrid Minimally Invasive Esophagectomy	0	1	-0.5914	0.4417
surgical_access	Total Minimally Invasive Esophagectomy	0	1	1.329	0.6405
surgical_technique	Ivor-Lewis	0	1	0.6747	0.5148
surgical_technique	Left thoracoabdominal	0	1	0.5022	0.8392
surgical_technique	McKeown (3-stage)	0	1	0.1326	0.5429
ngender	Female	0	1	-0.6177	0.3962
age_yrs		0	1	0.0166	0.0197

Odds Ratio Estimates

Effect	Dental problems	Point Estimate	95% Wald Confidence Limits	
complications 1 vs 0	0	1.098	0.524	2.299
years_since_surgery	0	1.014	0.817	1.257
neoadjuvant_therapy Yes vs No	0	1.432	0.631	3.252
surgical_access Hybrid Minimally Invasive Esophagectomy vs Total Open Esophagectomy	0	0.554	0.233	1.316
surgical_access Total Minimally Invasive Esophagectomy vs Total Open Esophagectomy	0	3.777	1.076	13.255
surgical_technique Ivor-Lewis vs Transhiatal	0	1.963	0.716	5.385
surgical_technique Left thoracoabdominal vs Transhiatal	0	1.652	0.319	8.56
surgical_technique McKeown (3-stage) vs Transhiatal	0	1.142	0.394	3.309

ngender Female vs Male	0	0.539	0.248	1.172
age_yrs	0	1.017	0.978	1.057

The LOGISTIC Procedure

Model Information	
Data Set	WORK.C30_OG25A
Response Variable	Hiccups
Number of Response Levels	
Model	generalized logit
Optimization Technique	Newton-Raphson

Analysis of Maximum Likelihood Estimates

Parameter		Hiccups	DF	Estimate	Standard Error
Intercept		0	1	3.7507	1.6328
complications	1	0	1	0.1783	0.3487
years_since_surgery		0	1	0.0622	0.1094
neoadjuvant_therapy	Yes	0	1	0.2774	0.4029
surgical_access	Hybrid Minimally Invasive Esophagectomy	0	1	0.6466	0.5809
surgical_access	Total Minimally Invasive Esophagectomy	0	1	-0.3741	0.3854
surgical_technique	Ivor-Lewis	0	1	-0.7146	0.6465
surgical_technique	Left thoracoabdominal	0	1	-0.6167	0.956
surgical_technique	McKeown (3-stage)	0	1	-1.081	0.6713
ngender	Female	0	1	0.1438	0.4201
age_yrs		0	1	-0.0116	0.0193

Odds Ratio Estimates				
**Effect**	**Hiccups**	**Point Estimate**	**95% Wald Confidence Limits**	
**complications 1 vs 0**	**0**	1.195	0.603	2.367
**years_since_surgery**	**0**	1.064	0.859	1.319
**neoadjuvant_therapy Yes vs No**	**0**	1.32	0.599	2.907
**surgical_access Hybrid Minimally Invasive Esophagectomy vs Total Open Esophagectomy**	**0**	1.909	0.611	5.96
**surgical_access Total Minimally Invasive Esophagectomy vs Total Open Esophagectomy**	**0**	0.688	0.323	1.464
**surgical_technique Ivor-Lewis vs Transhiatal**	**0**	0.489	0.138	1.738
**surgical_technique Left thoracoabdominal vs Transhiatal**	**0**	0.54	0.083	3.515
**surgical_technique McKeown (3-stage) vs Transhiatal**	**0**	0.339	0.091	1.265
**ngender Female vs Male**	**0**	1.155	0.507	2.631
**age_yrs**	**0**	0.988	0.952	1.027

Wald Chi-Square	Pr > ChiSq
0.1082	0.7422
7.0503	0.0079
2.5854	0.1079
2.6344	0.1046
0.2308	0.6309
4.3863	0.0362
3.1929	0.074
6.0776	0.0137
2.2556	0.1331
7.3965	0.0065
2.5833	0.108

Wald Chi-Square	Pr > ChiSq

4.098	0.0429
0.5287	0.4672
5.9783	0.0145
2.3573	0.1247
2.3657	0.124
1.4392	0.2303
0.0753	0.7838
0.0037	0.9518
0.5437	0.4609
1.126	0.2886
11.1217	0.0009

Wald Chi-Square	Pr > ChiSq
1.0268	0.3109
0.9371	0.333
0.7501	0.3864
1.8433	0.1746
0.0868	0.7683
0.5336	0.4651
0.6091	0.4351
1.4141	0.2344
0.5838	0.4448
1.5524	0.2128
3.9794	0.0461

Wald Chi-Square	Pr > ChiSq
0.1714	0.6788
0.1621	0.6873
0.2642	0.6072
0.0007	0.9788
0.4029	0.5256
0.7679	0.3809
3.285	0.0699
0.0712	0.7896
0.0586	0.8087
0.9878	0.3203
1.444	0.2295

Wald Chi-Square	Pr > ChiSq
2.9805	0.0843
0.1393	0.709
9.5997	0.0019
0.37	0.543
1.1399	0.2857

0.055	0.8146
14.3025	0.0002
2.7153	0.0994
11.8744	0.0006
1.6865	0.1941
4.71	0.03

Wald Chi-Square	Pr > ChiSq
0.3241	0.5691
0.4651	0.4952
2.719	0.0992
0.0485	0.8257
1.2526	0.2631
0.0284	0.8663
3.0339	0.0815
1.082	0.2983
2.2871	0.1305
1.6849	0.1943
0.353	0.5524

Wald Chi-Square	Pr > ChiSq

0.1043	0.7467
0.9077	0.3407
2.0551	0.1517
0.8551	0.3551
0.303	0.582
0.5117	0.4744
0.4917	0.4832
0.0895	0.7648
0.9837	0.3213
3.5822	0.0584
3.9354	0.0473

Wald Chi-Square	Pr > ChiSq
0.415	0.5194
1.2132	0.2707
3.1617	0.0754
0.6323	0.4265
0.9374	0.333
0.0061	0.9375
0.1036	0.7475
1.8246	0.1768
0.0699	0.7915
3.9962	0.0456
8.556	0.0034

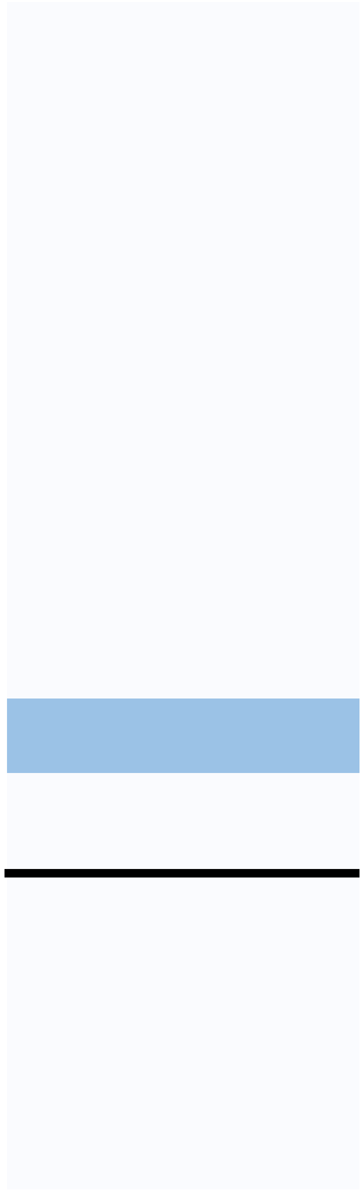

Wald Chi-Square	Pr > ChiSq
1.2957	0.255
0.8403	0.3593
3.5409	0.0599
0.0981	0.7541
4.2013	0.0404
5.9447	0.0148
1.4697	0.2254
0.6351	0.4255
0.3096	0.578
8.8068	0.003
14.246	0.0002

Wald Chi-Square	Pr > ChiSq
2.2776	0.1313
1.838	0.1752
2.0061	0.1567
0.6781	0.4102
3.78	0.0519

0.4786	0.4891
3.0078	0.0829
0.153	0.6957
1.0026	0.3167
2.5038	0.1136
5.4794	0.0192

Wald Chi-Square	Pr > ChiSq
0.3761	0.5397
0.8621	0.3532
0.0702	0.791
0.0001	0.9908
1.7569	0.185
0.0322	0.8575
3.7009	0.0544
6.865	0.0088
3.2892	0.0697
9.3302	0.0023
24.5409	<.0001

Wald Chi-Square	Pr > ChiSq

0.6602	0.4165
1.6699	0.1963
0.0279	0.8673
1.2192	0.2695
1.4549	0.2277
1.4516	0.2283
3.8974	0.0484
4.0836	0.0433
4.7772	0.0288
15.1706	<.0001
21.4839	<.0001

Wald Chi-Square	Pr > ChiSq
0.1393	0.7089
1.8756	0.1708
0.6506	0.4199
2.789	0.0949
0.5891	0.4428
2.9156	0.0877
0.0012	0.9727
0.382	0.5365
0.4657	0.495
1.932	0.1645
3.5517	0.0595

Wald Chi-Square	Pr > ChiSq
3.3778	0.0661
0.9946	0.3186
0.2994	0.5842
2.2448	0.1341
1.0751	0.2998
0.0014	0.97
0.6315	0.4268
0.6274	0.4283
0.1354	0.7129
5.2695	0.0217
19.1826	<.0001

Wald Chi-Square	Pr > ChiSq
0.0887	0.7658
0.0845	0.7713
0.2342	0.6284
0.2323	0.6298
3.3778	0.0661

3.8828	0.0488
1.2769	0.2585
0.1033	0.7479
0.2753	0.5998
5.3826	0.0203
10.2269	0.0014

Wald Chi-Square	Pr > ChiSq
0.4093	0.5223
0.885	0.3468
0.0082	0.9277
0.4569	0.4991
0.0081	0.9285
2.9928	0.0836
0.0293	0.8642
1.8849	0.1698
0.0172	0.8957
4.9288	0.0264
9.4207	0.0021

Wald Chi-Square	Pr > ChiSq

0.4117	0.5211
0.65	0.4201
0.2034	0.652
1.018	0.313
0.1965	0.6576
0.002	0.9641
0.4248	0.5145
0.7665	0.3813
0.1273	0.7212
4.8743	0.0273
11.9552	0.0005

Wald Chi-Square	Pr > ChiSq
4.3784	0.0364
0.0348	0.852
0.0661	0.797
2.3442	0.1257
0.29	0.5902
3.5812	0.0584
0.9166	0.3384
0.0573	0.8109
1.7579	0.1849
2.3224	0.1275
0.0182	0.8928

Wald Chi-Square	Pr > ChiSq
0	0.9972
1.7627	0.1843
0.2868	0.5923
3.3091	0.0689
0.0026	0.959
3.1122	0.0777
1.2426	0.265
0.0009	0.9759
0.0317	0.8588
1.1471	0.2842
2.9348	0.0867

Wald Chi-Square	Pr > ChiSq
0.0197	0.8885
0.0011	0.9737
0.117	0.7323
0.1658	0.6839
0.1526	0.6961

0.4846	0.4863
0.0865	0.7687
3.4086	0.0649
0.0008	0.9775
4.8134	0.0282
9.1098	0.0025

Wald Chi-Square	Pr > ChiSq
1.4192	0.2335
1.1209	0.2897
0.1947	0.659
2.0757	0.1497
0.437	0.5086
0.0872	0.7678
4.0943	0.043
2.1029	0.147
0.64	0.4237
1.7994	0.1798
0.0073	0.9321

Wald Chi-Square	Pr > ChiSq

3.9826	0.046
0.133	0.7154
0.3311	0.565
5.5114	0.0189
0.2879	0.5916
0.4355	0.5093
0.0056	0.9402
0.1335	0.7149
0.0001	0.9908
1.5738	0.2097
11.6595	0.0006

Wald Chi-Square	Pr > ChiSq
0.3946	0.5299
0.441	0.5067
1.0228	0.3119
2.6308	0.1048
1.5361	0.2152
0.7523	0.3858
2.4196	0.1198
1.1409	0.2855
0.6357	0.4253
0.2607	0.6096
3.21	0.0732

Wald Chi-Square	Pr > ChiSq
0.0089	0.9248
1.4792	0.2239
0.0431	0.8355
0.031	0.8603
0.2476	0.6188
5.3877	0.0203
2.6327	0.1047
0.2546	0.6138
0.3325	0.5642
0.1167	0.7326
1.524	0.217

Wald Chi-Square	Pr > ChiSq
3.2332	0.0722
0.1598	0.6893
0.7509	0.3862
0.213	0.6444
1.7569	0.185

0.142	0.7063
0.2716	0.6022
0.7905	0.374
0.0044	0.9473
0.6517	0.4195
0.0315	0.8592

Wald Chi-Square	Pr > ChiSq
0.2984	0.5849
0.0244	0.8759
3.0745	0.0795
0.3365	0.5618
0.5469	0.4596
0.5801	0.4463
4.7443	0.0294
3.4427	0.0635
2.5423	0.1108
0.7079	0.4001
0.1362	0.7121

Wald Chi-Square	Pr > ChiSq

0.6621	0.4158
0.0612	0.8046
0.0152	0.902
0.7371	0.3906
1.7923	0.1806
4.3048	0.038
1.7178	0.19
0.3581	0.5495
0.0597	0.807
2.4312	0.1189
0.7109	0.3991

Wald Chi-Square	Pr > ChiSq
5.2768	0.0216
0.2616	0.609
0.3233	0.5697
0.4738	0.4912
1.239	0.2657
0.9422	0.3317
1.2215	0.2691
0.4161	0.5189
2.593	0.1073
0.1171	0.7322
0.3628	0.547
