# Supplementary material for: Patient-reported outcomes after oesophagectomy in the multicentre LASER study
Source: Br J Surg. 2021 May 11;108(9):1090–6. doi: 10.1093/bjs/znab124 (PMC10364861; doi:10.1093/bjs/znab124)
Supplement: znab124_Supplementary_Data [file znab124_supplementary_data.zip › Table S1_LASER symptom grading.docx]

**Table S1 Grading system for each LASER symptom according the patient-reported impact upon quality of life (QOL) and frequency of the symptom**

| **Symptom level** | **QOL impact and Frequency** |
| --- | --- |
| 0 | No symptom present |
| 1 | QOL impact = None |
| 2 | QOL impact = Some & Frequency = rarely / weekly |
| 3 | QOL impact = Some & Frequency = daily / multiple |
| 4 | QOL impact = Substantial & Frequency = rarely / weekly |
| 5 | QOL impact = Substantial & Frequency = daily / multiple |
