# Supplementary figures and images for: Patient-reported outcomes after oesophagectomy in the multicentre LASER study
Source: Br J Surg. 2021 May 11;108(9):1090–6. doi: 10.1093/bjs/znab124 (PMC10364861; doi:10.1093/bjs/znab124)

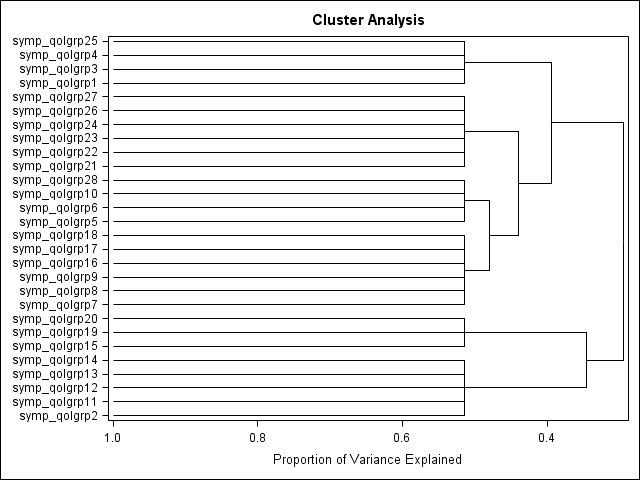

Supplement: znab124_Supplementary_Data [file znab124_supplementary_data.zip › Fig. S1_Cluster_Dendrogram.png]
